# Drone and ground-truth data collection, image annotation and machine learning: A protocol for coastal habitat mapping and classification

**DOI:** 10.1016/j.mex.2024.102935

**Published:** 2024-08-30

**Authors:** Kristina Øie Kvile, Hege Gundersen, Robert Nøddebo Poulsen, James Edward Sample, Arnt-Børre Salberg, Medyan Esam Ghareeb, Toms Buls, Trine Bekkby, Kasper Hancke

**Affiliations:** a Norwegian Institute for Water Research (NIVA), Økernveien 94, 0579 Oslo, Norway; b SpectroFly ApS, Markstien 2, 4640 Faxe, Denmark; c Norwegian Computing Center, Gaustadalléen 23a, 0373 Oslo, Norway

**Keywords:** Uncrewed/Unoccupied/Unmanned Aerial Vehicles (UAVs), RGB imagery, Multispectral imagery (MSI), Machine learning (ML), Habitat mapping, Coastal vegetation, Biodiversity, *In situ* data sampling, Macroalgae, Intertidal zone, Field data collection and drone image annotation for coastal habitat mapping

## Abstract

Aerial drone imaging is an efficient tool for mapping and monitoring of coastal habitats at high spatial and temporal resolution. Specifically, drone imaging allows for time- and cost-efficient mapping covering larger areas than traditional mapping and monitoring techniques, while also providing more detailed information than those from airplanes and satellites, enabling for example to differentiate various types of coastal vegetation. Here, we present a systematic method for shallow water habitat classification based on drone imagery. The method includes:

• Collection of drone images and creation of orthomosaics.
• Gathering ground-truth data in the field to guide the image annotation and to validate the final map product.
• Annotation of drone images into – potentially hierarchical – habitat classes and training of machine learning algorithms for habitat classification.

As a case study, we present a field campaign that employed these methods to map a coastal site dominated by seagrass, seaweed and kelp, in addition to sediments and rock. Such detailed but efficient mapping and classification can aid to understand and sustainably manage ecologically and valuable marine ecosystems.

## Graphical abstract

**Figure:**
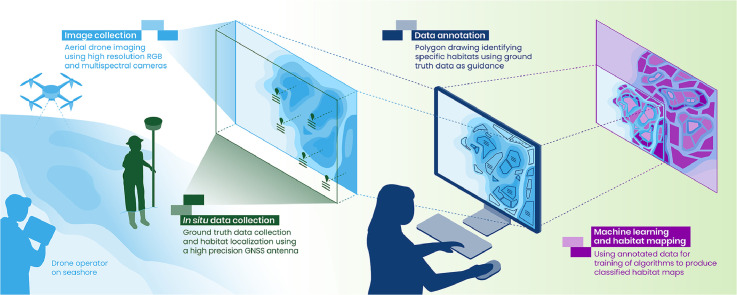

Specifications table

**Table:** 

Subject area:	Environmental Science
More specific subject area:	Coastal habitat mapping and classification
Name of your method:	Field data collection and drone image annotation for coastal habitat mapping
Name and reference of original method:	Not relevant
Resource availability:	Flying drones (UAVs) with RGB and/or MSI sensors; GPS positioning instruments (preferably high-precision GNSS systems); high-performance computer with Pix4D or other equivalent software; and ArcGIS Pro or other equivalent software.

## Method details

### Background information

Coastal vegetated habitats such as kelp forests, rockweed beds, seagrass meadows and tidal marshes, often called “blue forests”, cover vast areas around the globe. These habitats provide a multitude of ecosystem services, including as spawning, nursery, shelter and feeding areas for ecologically and economically important species, as carbon sink, as source of raw material, by preserving or improving water quality, and as shore protection during extreme weather events [[1], [2], [3], [4]]. These ecosystem services create the basis for several of the United Nations Sustainable Development Goals (SDGs), e.g., zero hunger (SDG #2), climate action (SDG #13), and life below water (SDG #14) [5].

The world's coastlines are under increasing pressure from urbanization and industrialization [6]. This, together with the effects of climate change, leads to degradation and loss of coastal habitats worldwide, with consequently dramatic declines in biodiversity and changes in ecosystem functioning [[7], [8], [9]]. To stop this development, we need to conserve and protect at a higher rate, as expressed by the Global Biodiversity Framework [10], with the aim to protect 30 % of Earth's lands, oceans, coastal areas, and inland water by 2030. To meet this ambitious goal, we must speed up the development of cost-effective and accurate methods to map and classify valuable marine areas.

Flying drones, or Uncrewed Aerial Vehicles (UAVs), are increasingly being used for different applications within coastal zone management, such as mapping and monitoring of species and habitats [[11], [12], [13]] and detecting marine litter and disaster management [14,15]. At the habitat level, and for species that are identifiable from a distance, the use of drones in coastal areas allows time- and cost-efficient mapping covering larger areas than traditional mapping and monitoring techniques such as underwater cameras and aquascopes [16]. Also, drone images provide more detailed information than those from airplanes and satellites, enabling the differentiation of various types of coastal vegetation based on individual spectral reflectance and structural patterns. E.g., flying at 100 m above ground, drones can collect images with a sub-centimeter ground sampling distance (GSD), which is at least 50 times better than what is available from commercial satellites (e.g., eos.com/products/high-resolution-images), and cover an area of up to ∼1 km^2^ per hour (rapidly increasing with the fast-developing technology).

The use of drone imaging in combination with sophisticated Artificial intelligence (AI) / machine learning (ML) algorithms, such as Convolutional Neural Networks (CNN) [17], may ultimately allow us to map and classify coastal habitats with minimal or no field data collection, relying instead on minimal ground-truth data collection for result validation. However, ground-truthing is still required when training new algorithms. The collection of accurate and georeferenced observations serves two purposes; 1) guide the annotation of drone images used as training data for ML algorithms, and 2) validate classified prediction maps resulting from the ML analyses.

In this protocol, we outline a systematic and standardized method to collect drone images and ground-truth data in coastal zones, and the subsequent annotation (identification of different habitat types and other features) of drone images to inform the ML algorithms for habitat classification. The ML analyses are just briefly described here and explained in more detail elsewhere [[18], [19], [20]]. This protocol is based on method improvements and experiences accumulated through numerous drone mission campaigns and subsequent data analyses during > 5 years, as part of the SeaBee research infrastructure for drone-based research, mapping, and monitoring (www.seabee.no). Although focused on the coastal zone, this protocol is largely applicable for freshwater, and even terrestrial field data collection.

### Drone image acquisition

#### Rules and regulations

All necessary authorizations and licenses must be in place before the collection of drone images can take place, and international and national rules and regulations must be considered (e.g., the European www.dronerules.eu). One should make a pre-flight check list of important points to consider before each mission (e.g., obstacles, restricted zones, safety zones, nature protection considerations, environmental conditions, inspection of equipment, etc.) – there are many useful sites on the internet (see also [21]).

#### Drones

**Fig. 1:**
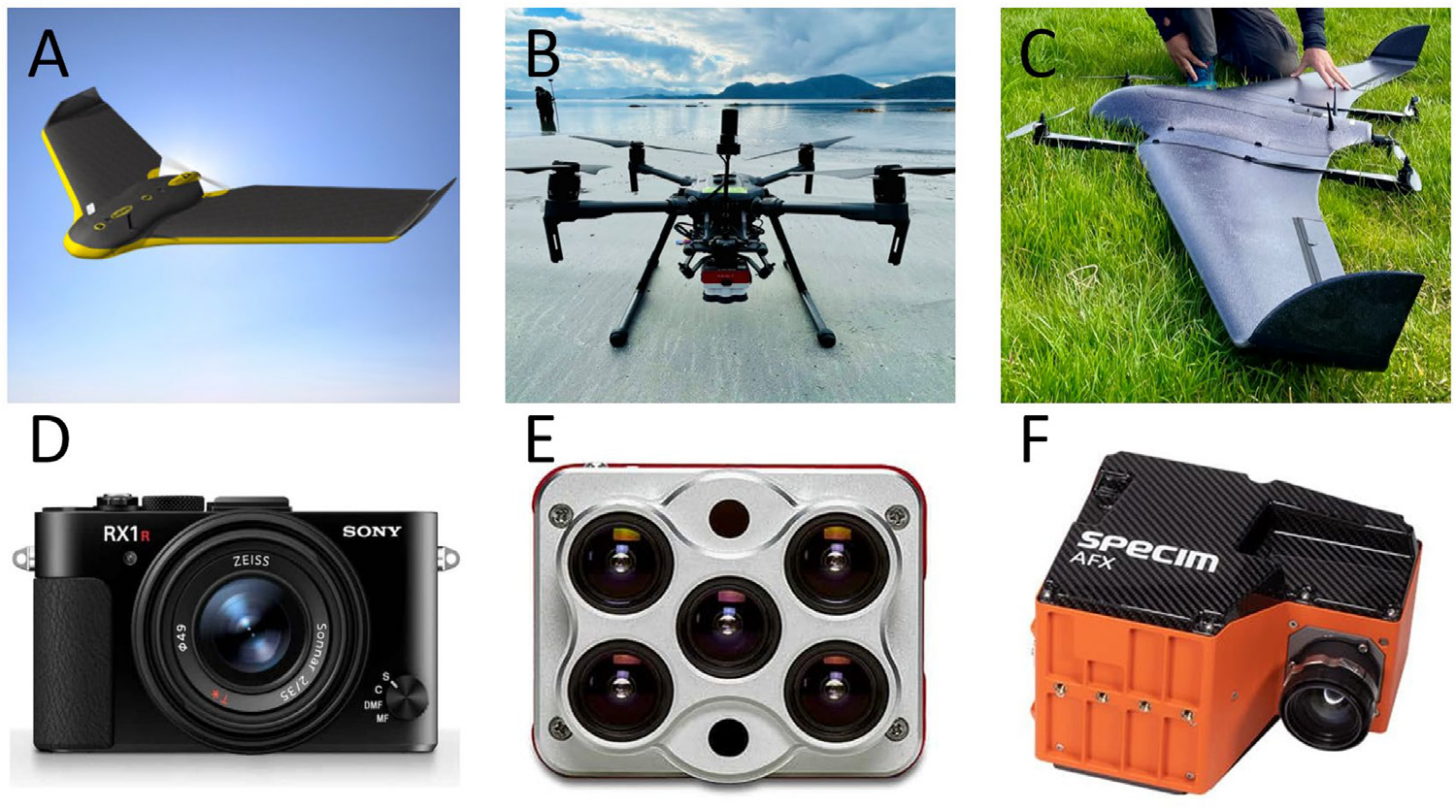
Examples of drone and sensor types commonly used for coastal habitat mapping: A) fixed-wing drone (eBee X mapping drone, AgEagle Aerial Systems); B) rotor drone (DJI Matrice 300, photo by SeaBee); C) vertical take-off and landing (VTOL) drone (DeltaQuad Pro, DeltaQuad, photo by SeaBee); D) Red, green and blue (RGB) sensor (Sony RXR II, Sony Group Corporation); E) multispectral imagery (MSI) sensor (MicaSense Altum, Ag Eagle Inc.); F) hyperspectral imagery (HSI) sensor (Specim AFX10, Specim, Spectral Imaging Ltd.)

There are three main types of flying drones that are used for coastal habitat classification. These are fixed-wing drones, rotor drones, and vertical take-off and landing (VTOL) drones (Fig. 1). The latter combines the benefits of the first two systems and uses rotors to lift a fixed-wing drone vertically for take-off and landing. These drone types serve somewhat different purposes, and their use depends on the scope of the campaign. Rotor drones are easily maneuvered, can fly at low speed, and stand still in the air. Fixed-wing drones, on the other hand, are more energy efficient, and typically have longer flight times, and are therefore often used for long-distance missions. However, most fixed-wing drones require a take-off and landing zone, which can be hard to find when working in the coastal zone and remote areas. VTOL drones combine the two, which particularly makes landing easier. Table 1 summarizes the applications, advantages and disadvantages of the different drones.

**Table 1:** List of equipment types (drones, sensors, and positioning systems) for coastal habitat mapping, and their applications and usefulness.

Equipment type	Application	Advantages	Disadvantages
**Drones for aerial survey**			
Rotor drones (e.g., DJI MATRICE 300 RTK)	• Detailed mapping	• Adjustable flight speed/stand-still • Slower speed allows higher spatial resolution in images • Large payload capacity • Low and high altitude • Vertical take-off and landing	• Limited areal coverage
Fixed wing drones (e.g., SenseFly eBee)	• Large scale mapping	• Energy efficient • Larger aerial coverage • Long distance	• Smaller payload capacity • Large landing zone (unless equipped with VTOL) • Susceptible to wind direction • Fixed flight speed • Risk of smearing at low altitudes
**Sensors (cameras) attached to the drone**			
Red, green and blue (RGB) sensors (e.g., SONY RX1RM2 42MP)	• Annotation and study site overview • Simple habitat classification	• True colors (as seen) • High pixel resolution	• Low spectral resolution and low sensitivity for species and habitat recognition • Not radiometrically correct (overlap between colors) • Pixel values are represented with a digital number, not surface reflectance
Multispectral imagery (MSI) sensors (e.g., MicaSense Altum 5band)	• Advanced/ detailed habitat classification • Pixel-wise thematic mapping	• Several images at specific wavelengths • Radiometrically correct color sensing • Enable comparison with spectral reflectance libraries	• Less suitable for annotation because of unnatural colors • Lower pixel resolution • Increased complexity
Hyperspectral imagery (HSI) sensors (e.g., Specim AFX10)	• Species detection, detailed habitat classification	• High spectral resolution • Can reconstruct true color images • Potential for physiological and ecological status assessment of marine vegetation	• Low light sensitivity • Low pixel resolution • Requires complex data handling and analyses • Creates large amounts of “non-essential” data • Heavy weight • Expensive
**Positioning system (Global Navigation Satellite System, GNSS)**			
Handheld GNSS (e.g., Garmin Montana 700)	• Ground-truthing	• Inexpensive • Pocket size • Robust (water/splash proof) • Immediate response time when taking a position	• Low spatial accuracy (∼2 m), i.e., ground-truth data can only be used as guidance
High precision GNSS (e.g., Leica GS18 T RTK Rover)	• Ground-truthing	• High spatial accuracy (< +/-2 cm) • Programmable • Ground-truth data can be used as annotation directly	• Requires correction signals (RTK) • Expensive • Large size • Not fully waterproof • Sometimes long response time for taking positions (when low signal coverage/quality)

#### Sensors

Drones can be equipped with different types of sensors. Three types of optical sensors (i.e., cameras) are typically used for coastal habitat mapping; red, green and blue (RGB), multispectral imagery (MSI) and hyperspectral imagery (HSI) sensors (Fig. 1). These sensors are associated with advantages and disadvantages that are summarized below and in Table 1. Thermal sensors (sensitive to the infrared spectrum outside the visual range) and Light Detection And Ranging (LIDAR) sensors are also becoming increasingly available for drone applications, but are not discussed further here.

**Fig. 2:**
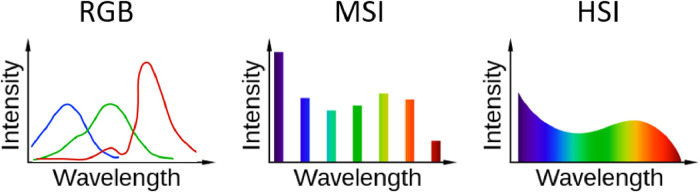
Comparison of spectral sampling in red, green and blue (RGB), multispectral imaging (MSI), and hyperspectral imaging (HSI) sensors. Modified from: Lucasbosch, CC BY-SA 4.0, via Wikimedia Commons.

RGB images are obtained using a traditional digital camera. The sensor captures images in three color channels (red, green, blue), and the color of each pixel in the resulting image is a combination of different intensities of these three primary colors. Values typically range from 0 to 255 (or higher depending on sensitivity, i.e., 2^x^ where x determines the sensitivity), where 0 is the absence of color and 255 is the maximum intensity of that specific color. For example, a pixel with an RGB value of (255, 0, 0) would be pure red. RGB sensors typically have overlapping spectral sensitivity, meaning that, the green channel is sensitive to green light but is also partly sensitive to blue and red light, and the same is true for the blue and red channels (Fig. 2). This hampers a specific quantification of the light in separate colors, a problem that MSI sensors are designed to overcome.

MSI sensors capture several images in parallel across the electromagnetic spectrum, each sensitive only to specific wavelength ranges or bands (often between 4 and 6 bands). Typically, MSI sensors are sensitive to radiation across a band width of 10 to 60 nm and are not sensitive to light outside this range (Fig. 2). Often, red, green, and blue are among these, but MSI sensors may additionally capture images in other wavelengths, including those in the near-infrared (NIR) range or at longer wavelengths outside the visual spectrum (i.e., 400-700 nm). The specific spectral bands captured may vary with the intended application of the sensor. E.g., vegetation has strong absorption from pigments in the visible spectrum (especially in blue and red), strong reflection and transmission from the intercellular walls in the NIR, and absorption from water in the short-wave infrared (SWIR, [22]). The most common spectral bands for coastal habitat mapping include blue (around 475 nm), green (around 560 nm), red (around 668 nm), red-edge (around 717 nm), and NIR (around 842 nm). Many commercially available MSI sensors for drones mimic the spectral sensitivity of MSI sensors on satellites (e.g. Sentinel 2, [23]). In contrast to RGB images, MSI sensors collect quantitative spectral irradiance data, as the number of photons (i.e., photon flux density) at specific wavelengths are recorded by the sensor. The MSI signal can thus be corrected for the background light field (the downwelling irradiance) and thereby provide spectral reflectance data of specific objects or habitats independent of the solar intensity (the spectral irradiance). This feature is elevating MSI (and HSI sensors, see below) relative to RGB sensors for habitat mapping, as calibrated data can be quantitatively compared to databases of spectral reflectance (optical fingerprints) for specific vegetation, species, or anthropogenic objects such as plastics.

An HSI sensor captures light at hundreds of different wavelengths and is thus comparable to an MSI sensor but has higher spectral resolution (often 1-3 nm, Fig. 2). HSI sensors scan the environment in a so-called “push-broom” manner (i.e., along-track scanning, much like an office document scanner). In consequence, the HSI sensors do not collect individual images, but a specific duration of scanning can be combined to form images in which each pixel contains information from the entire electromagnetic spectrum, within a given range [24]. HSI therefore provides a detailed spectral profile of a specific species or habitat type. Unlike MSI, which captures a limited number of bands, HSI captures spectral data in a continuum (Fig. 2), allowing for a more detailed analysis of spectral properties and proving a tool for bio-optical taxonomic recognition [25], alike an optical fingerprint. The spectral sensitivity of HSI sensors available for drones typically ranges from 400 to 1000 nm or ∼1000 to ∼2000 nm.

When experts annotate based on recognition by the human eye, RGB images are preferable as they offer a realistic, true color impression of the species or habitat. Moreover, RGB images provide a useful birds-eye perspective of the study site. For ML analyses, however, MSI and HSI are superior since they contain extra channels of information, including those in the non-visible spectrum, and can provide qualitative-corrected optical data through calibration. Typically, within the same camera weight class, RGB sensors deliver higher spatial resolution (pixels per cm) compared to MSI sensors. MSI sensors, due to their more complex nature (several sensors compared to one), have higher costs and produce larger data volumes. HSI, in turn, captures information across a significantly broader spectrum than both RGB and MSI, making it a promising choice for detailed habitat classification, including better differentiation of vegetation types, potentially assessing habitat health status, and identifying other features of interest [26,27]. Still, the sheer complexity of HSI data collection (scanning one transect at the time, requiring post-processing stitching), combined with immense volume of data created and elevated computational demands, restrain its large-scale adoption for coastal habitat mapping (but see, e.g. [28]). Nevertheless, HSI sensing from satellites is emerging as a promising tool for ocean and coastal observations [29].

#### Planning and pre-programming of the fly path

Areal mapping comes with the trade-off between the areal coverage (size) and the spatial resolution (detail) in the resulting drone image. For instance, for a 30 min RGB sensor flight, one can map an area of 0.20 km^2^ with a GSD (image resolution) at 0.75 cm/pixel, or an area of 0.40 km^2^ at a resolution of 1.5 cm/pixel. The difference lies in the flight altitude, 60 m versus 120 m in this case (at a flight speed of 8 m/s, with 75 % frontal and 75 % sideway overlap). Roughly speaking, when the altitude is doubled, the area covered also doubles, while the GSD decreases correspondingly. If one additionally increases the speed and decreases the image overlap, the mapping efficiency can be increased up to three times with a doubling of the altitude, however at the cost of image quality and orthomosaic stitching performance.

Importantly, different sensors have varying pixel resolutions, which will result in different GSD, even when flown at similar altitudes. For instance, an MSI sensor might have half the pixel resolution of an RGB sensor. To obtain similar GSD, the MSI sensor would need to be flown at a lower altitude. Alternatively, a comparable GSD can be obtained from two different sensors (or altitudes) during post processing by resampling the higher resolution images to match those with the lowest resolution.

In more detail, area mapping capacity is determined by flight altitude, flight speed, and image overlap, but the obtainable image resolution also depends on sensor specifications. These four factors can be adjusted by the operator with some limitations (e.g. flight regulations, terrain features, hardware setup, and minimum speed for fixed and VTOL wing drones). The trigger interval and the shutter speed are two of the sensor specifications that can limit the image quality, particularly when attempting to achieve high overlap at low altitudes and at high speed. The lower the flight altitude, the lower the drone speed or the faster the shutter speed needs to be to avoid “image smearing” or “motion blurring”. Online calculators and drone mission planning tools can be helpful when balancing area covered, spatial resolution, flight altitude, sensors specifications, along with battery capacity and local terrain features (e.g., www.pix-pro.com/blog/post/photogrammetry-calculator). Drone manufacturers typically include flight planning tools as a part of their mission planning software, and various commercial alternatives are used among drone operators for advanced flight mission planning (e.g. UgCS Flight Planning by SPH Engineering). Considering the multitude of cameras available for drone mapping, it is not feasible to provide a universal set of recommendations for camera settings here, and we instead encourage users to follow the specific manufacturers’ guidelines.

For habitat mapping, drones should ideally be pre-programmed to fly in automatic “lawnmower” or chessboard grid patterns, which ensures that images are captured sequentially, facilitating systematic and efficient image collection and subsequent image processing. Some degree of overlap between images is necessary to secure high quality orthorectification and (for most software) to generate orthomosaics (large map products resulting from the combination and orthorectification of many smaller images). The optimal overlap for intertidal and shallow water habitat mapping is typically 80 % or more. This is due to the number of recognizable features often being much fewer than on land, depending on the environmental conditions and nature of the mission area. A high degree of overlap (e.g., 80 % forward and 70 % sideways), will ease the stitching process and thus improve the quality of the resulting orthomosaic, but reduce the total area covered per unit time.

#### Georeferencing

**Fig. 3:**
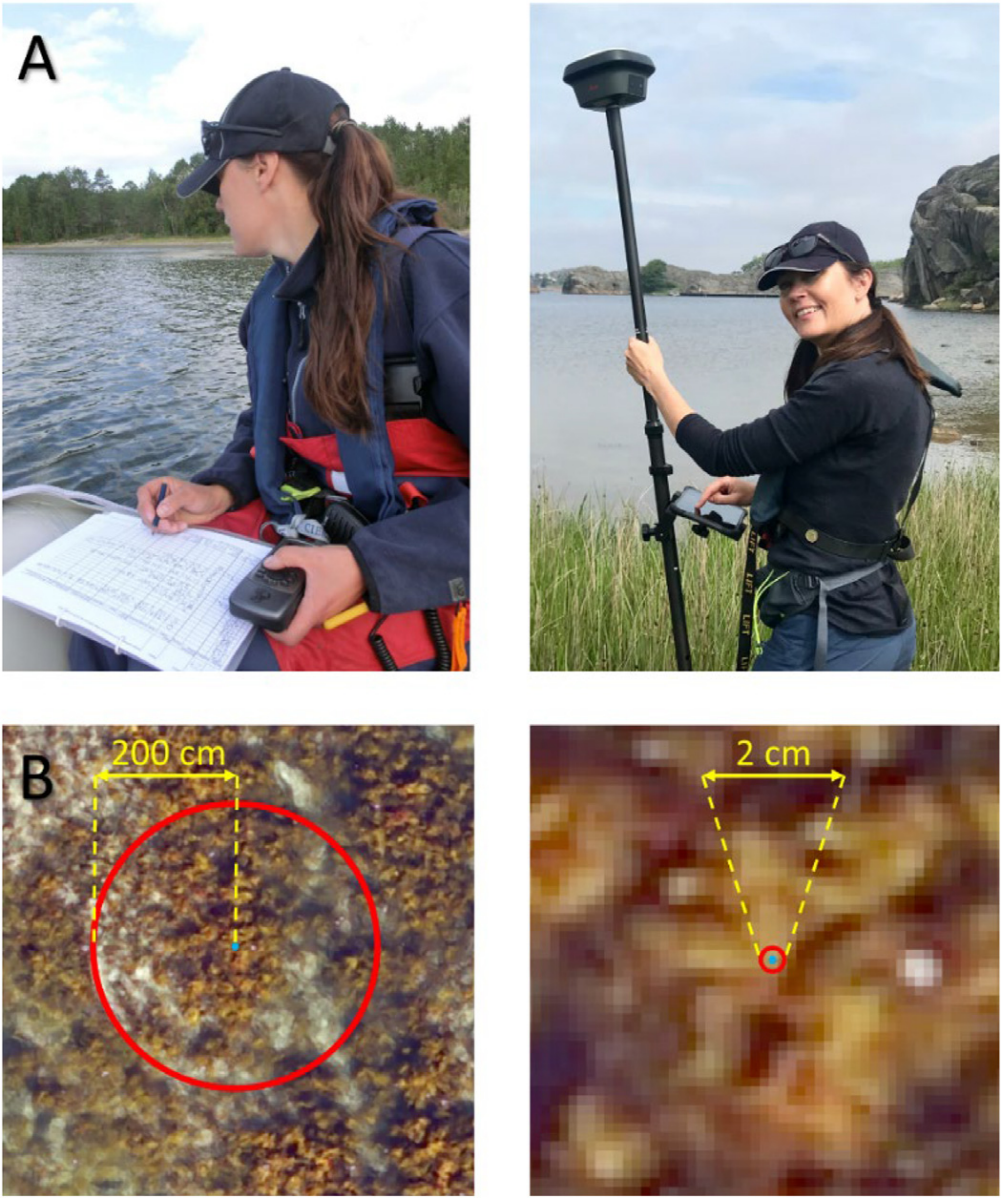
A) Two types of instruments used for georeferencing of ground truth data during fieldwork, i.e., a low-precision handheld GPS (left) and a high-precision GNSS (right). B) Close-up drone images of kelp (*Laminaria hyperborea*) patches on sand bottom, illustrating a ground-truth observation (blue dot) and the positioning accuracy stemming from the two instruments in A (red circles). Ground Sample Distance (GSD) = 1.5 cm. Photos by SeaBee (kelp photos from Vega, Norway, 18.08.2022).

To provide useful data products, both drone images and ground truth data must be georeferenced, either using regular GPS sensors or high-accuracy Global Navigation Satellite System (GNNS) sensors (Fig. 3 and Table 1).The accuracy of these systems depends on signal quality at the time of collection, which in turn depends on geographical location of the receiver, time of day, and the signal provider. Regular GPS receivers can be trusted to provide an accuracy of around 2 m (but occasionally better), while high-accuracy GNSS receivers typically have a trusted accuracy of less than 3 cm (often <1 cm) in the horizontal directions (Fig. 3 and Table 1, [30]). The vertical accuracy is approximately three times coarser than the horizontal (due to the triangulation of satellite data), which is relevant for 3D terrain modelling, water depth measurements and volume estimations [21,31]. The accuracy required for a specific dataset depends on its application; to stack several images and other data products on top of each other (e.g., drone-based orthomosaics, ground truth data, annotation data, and final habitat classification data), high geospatial accuracy is needed; while for single data products (e.g., simple orthomosaics for costal overviews), high-accuracy positioning is less critical.

For accurate (cm scale) georeferencing, images must be tagged with high-accuracy positioning data either in-flight with Real Time Kinematic (RTK) systems or post-flight using Post Processed Kinematic (PPK) techniques. Achieving this level of positioning accuracy, requires that the drone carries a GNSS RTK receiver able to connect to multiple satellites and a base station (for RTK) or access to data from reference stations/virtual stations (for PPK) [32]. If only regular GPS signals are available for the drone navigation system, placement of ground control points (GCPs) can enhance the absolute positioning accuracy of orthomosaics through post-processing. GCPs are distinct and accurately georeferenced points marked on the ground, ideally at sub-centimeter spatial resolution. A GCP can be any physical object, for instance, a wooden or painted cross on the ground that can be recognized in drone images (in the latter case, one should be sure to use environmentally friendly paints). The GCPs are georeferenced in the field using a high-precision GNSS instrument with < 3 cm accuracy. The number of GCPs needed for accurate positioning depends on the size and typography of the site, but a minimum of five GCPs is typically recommended for small-scale drone missions [31,33]. In addition to georeferencing orthomosaics generated without RTK or PPK, high-precision GCPs can serve to quantify the absolute accuracy of the RTK/PPK positioning, by evaluating the distance (x, y, z) between the GCP positions in the orthomosaic and on measured *in situ*.

#### Environmental conditions

Environmental conditions and the weather influence drone and sensor performance and hence the quality of the collected images. In practice there is rarely an ideal time to fly, and mission planning will often focus on balancing different pros and cons towards the mission aims, and avoiding conditions that will be detrimental to image quality. Below, we discuss the most important environmental conditions to consider when planning drone missions in the field. Some are predictable and can be planned for in advance (e.g., tide and sun angle) while others are more unpredictable (e.g., temperature, wind, waves, cloud cover and rain). In addition to considering image quality, one must avoid flying during conditions that can damage the equipment or pose safety risks (e.g. windspeeds higher than 6-10 m/s, depending on the pilot's experience, temperature extremes [<0°C/>40°C] that can damage or reduce the drone's performance, and fog or rain that reduce the pilot's line of sight and can damage the drone). Several safety checklists for drone flying are available online, and the drones’ user manuals contain information on recommended flight conditions. Below, we focus on aspects that influence the quality of the collected image data.

**Fig. 4:**
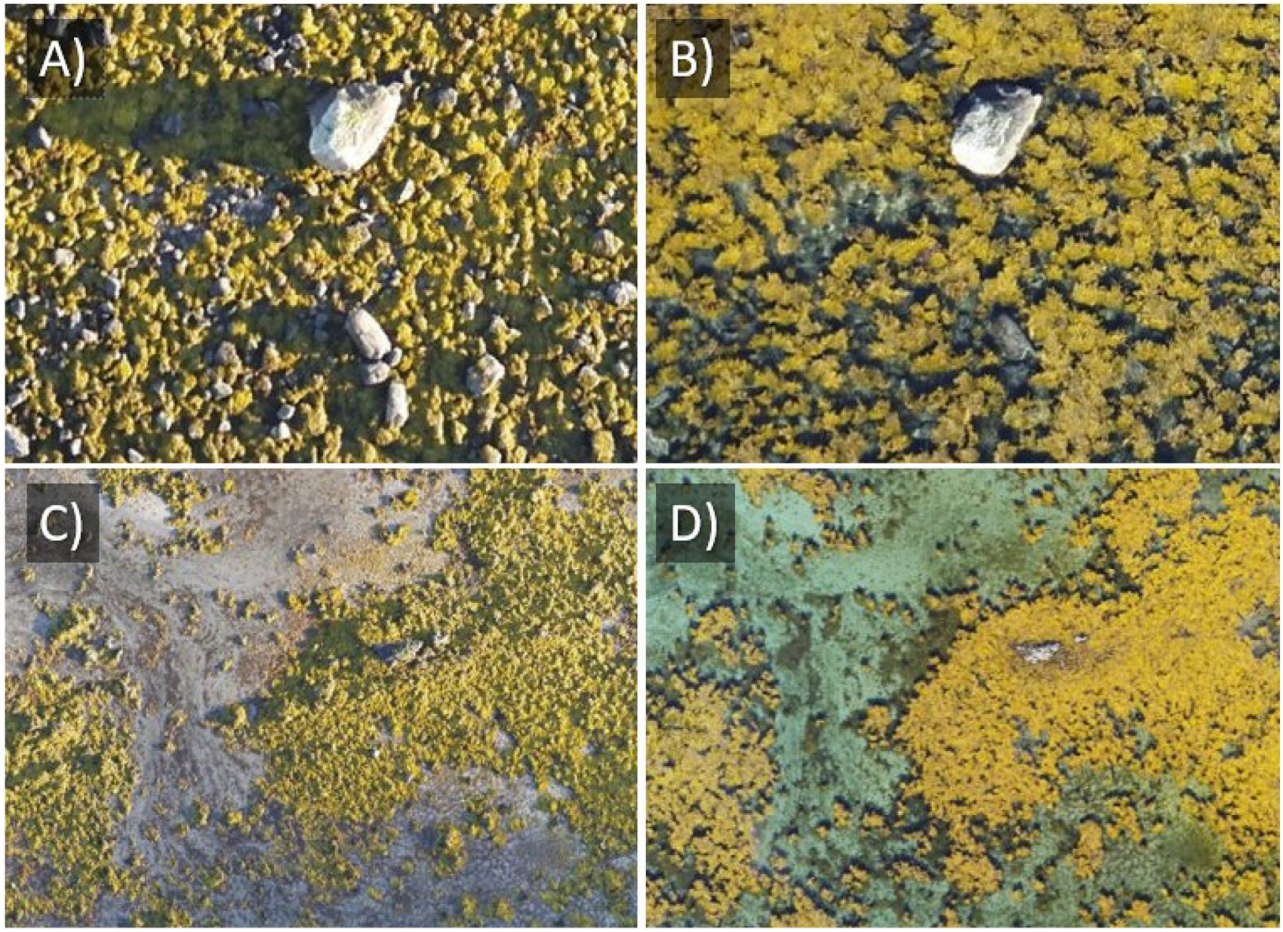
Details from drone images that show A) long shadows on images taken early in the day (07:30) when the solar angle is low, and B) short shadows later in the day (13:00) when the sun is high on the sky; and macroalgae at C) low versus D) high tides.

**Sunlight and solar altitude angle**: Direct sunlight with drifting clouds can create unfavorable, alternating light conditions, therefore, light and homogeneous overcast conditions are often preferred. Diffuse light conditions help minimize shadows and provide a more uniform illumination both above and below water [34], which improves the quality of image stitching and analysis. At early or late hours during the day, the low solar altitude angle (also called solar elevation angle, i.e., the angle between the horizon and the sun's position in the sky, hereafter solar angle) will result in long shadows in drone images, as illustrated in Fig. 4 for intertidal macroalgae. In general, it is recommended that the solar angle is >20° for the best possible underwater light field. At a lower solar angle, in particular <10°, the fraction of solar radiation that travels below the air-water interface and eventually returns from the sea surface is dramatically decreased [34], which reduces the signal-to-noise ratio. However, at higher solar angle, the fraction of the solar light that is reflected at the sea surface relative to at the seafloor increases, which also reduces the signal-to-noise ratio. In particular, flying at around solar noon may result in bright reflective spots of sunlight (sun glints) reaching the optical sensor. This causes overexposure of the sensor and hampers data collection. Sun glints and occasional overexposure can be partly corrected for during post-processing [35], but to avoid sun glints, it is recommended to collect images when the solar angle is <35-40° [21,36,37]. For mapping and surveying purposes, the ideal solar angle for collecting drone images normally occurs from mid-morning to mid-afternoon, depending on latitude and time of year. Considering the solar angle is particularly important at higher latitudes, for example, some MSI sensors equipment with incident downwelling light sensors require a minimum 30º solar angle, meaning that in high-and low-latitude regions, light correction will not work properly during winter or early morning/evening. The solar angle can be calculated using online tables, e.g., https://gml.noaa.gov/grad/solcalc/azel.html. If the drone imaging must be performed during periods when the sun is high in the sky, the solar azimuth should also be considered (i.e., the angle between the sun's position and a due north line). A flight direction directly away from the sun is recommended to best avoid sun glints, described in more details by [21].

**Water transparency**: In addition to solar angle, water transparency is likely the most important environmental factor influencing drone image quality when mapping coastal habitats submerged in water [37]. High water transparency (low light attenuation) allows sunlight to penetrate through the water column, return from the sea floor and be detected by the drone's sensors. Water column light attenuation is determined by the concentration of phytoplankton, colored dissolved organic matter (cDOM) and suspended particulate matter (SPM), in addition to the light absorption by water itself [22]. Therefore, periods with high concentrations of phytoplankton, typically during spring and potentially fall blooms, should be avoided, or carefully considered in mission planning. Furthermore, one should avoid collecting images closely after heavy rainfall or snow thawing. Coastal waters typically become more turbid after heavy rainfall, as rivers discharge organic (cDOM) and inorganic (SPM) material. Freshwater input can also impact the optical properties of surface water through the formation of a freshwater layer on top of saline, marine waters. The concentration of phytoplankton, cDOM, and SPM can be estimated *in situ* using optical sensors or derived from laboratory analysis of water samples. *In situ* sensors can be handheld (point measurements) or be deployed from small boats or Uncrewed Surface Vehicles (USV) to provide spatial coverage. As a low-tech alternative, water transparency can be measured in the field using a Secchi disk. The Secchi depth is an approximate measure of water transparency based on the depth at which an observer loses sight of a physical disk that is lowered into the water. Albeit simple, it provides a good proxy for how deep benthic habitats can be observed using airborne optical sensors. Ideally, a Secchi depth >5-10 m is desirable, but good data can be collected at lower values when mapping at shallow water (e.g., >5 m Secchi depth for seagrass mapping [37]) or in the intertidal.

**Cloud coverage**: Cloud coverage is *per se* not a major challenge for drone image collection, since drones, in contrast to satellites, usually operate below the cloud layer. As described above, a homogenous cloud cover can be preferable as it diffuses the solar radiation and reduces shades, sun glints and glare, and can improve stitching over deeper areas of water. In fact, very low (<10 %) or very high (>90 %) cloud cover is preferable, as it creates more consistent reflectance conditions [37]. But cloudy conditions can decrease the light penetration through water, restricting the depth of the seafloor that is visible in the images, and may also result in images with somewhat less contrast and less intense colors, making it harder to distinguish details. Fluctuating or patchy cloud cover combined with strong sunlight are particularly challenging conditions for drone data collection due to variable and inconsistent light, shading and color conditions in the imagery.

**Tidal level**: For coastal habitat mapping in areas with tidal differences, one should aim to capture the images at low tide (± 2 h), when the coastal vegetation is more exposed. This allows the sensors to “see” as far as possible into the water, providing a more detailed view of the seafloor (Fig. 4). Moreover, the positioning of marine vegetation can be somewhat shifted when submerged in water (e.g., from lying flat when dry to standing erect in the water, Fig. 4), challenging the intercomparison of images taken at different time points and the use of ground truth data for image annotation. As shown in Fig. 4, the tidal level also influences the optical signature of marine vegetation. In general, there are two low tides each day, with a period of approximately 12 h and 25 min between each, and the tidal levels for a region and time of year can be found using online tables (e.g., www.tideschart.com). To meet the lowest tides possible in an area, one may also want to plan missions during periods of spring tides. Air pressure will also impact the water level, and in areas of small tidal differences, the air pressure and wind can have significant – and less predictable – impact (e.g., as the air pressure drops, the water level rises, and vice versa, see [38]).

**Waves and wind:** Waves can obscure features on the seafloor by causing reflections and glare on the surface of the water, which can interfere with the drone's optical sensors [36]. This can result in overexposed or underexposed images, or images that are washed out and lack contrast. Furthermore, the wave movements can result in images that are out of focus or have motion blur. Wave heights >1 m should be avoided, especially if they have foamy crests, which may be created already at wind speeds around 4 m/sec (level 3 at Beaufort scale). On the other hand, a completely flat sea surface will reflect a larger fraction of the light than under light wind conditions (around 2-4 m/s), the latter resulting in a larger fraction of the light reaching the sea floor and being reflected back to the sensor.

**Rain and humidity**: Even though waterproof drones are emerging, one should avoid collecting data in heavy rain to prevent water on the lens. Light rain and/or high air humidity can also cause droplets on the camera lens and lead to distortion and blurring of the images.

**Temperature:** Finally, in addition to influencing battery capacity and flying conditions, the air temperature can influence image quality. At high temperatures, there may be heat haze in the air, which can cause optical distortion affecting the images. On the contrary, low temperatures may increase the risk of condensation on the lens, which can also affect the quality of the images.

In sum, fieldwork planning should be done in accordance with hours of daylight, tide table, and weather forecasts, but also by considering the actual conditions experienced in the field. Optimal conditions for image collections with drones include low tide, clear and calm water, calm wind, medium solar elevation, and homogenous cloud conditions that remain stable over the duration of the drone missions. While it is difficult to make broad generalizations about the suitability of drone-based imagery for coastal habitat classification for different regions, the method performs better and at greater depths in areas with higher water clarity. This includes tropical and oligotrophic regions and outer coastal areas, where concentrations of chlorophyll, organic, and inorganic matter are often lower. Notably, kelp forests were recently mapped down to a depth of 10 m around exposed islands in Northern Norway [39], a depth similar to what is considered feasible for coral reef mapping in clear waters [40]. Conversely, mapping beyond 2-3 m depth becomes challenging under less ideal conditions, such as reduced water clarity due to clouds, wind and sun glints [41]. Since both aquatic vegetation and drone sensors are limited by light penetration, the depth to which drone images can effectively “see” is proportional to the depth extent of the submerged vegetation. Therefore, the fraction of submerged vegetation observable from drones with image sensors is larger for species with high light requirements, such as seagrass [42,43], and smaller for species with low light requirements, such as microalgae and coralline algae [42,44].

#### Post-processing of drone images

After collecting images in the field, post-processing is required to obtain high-quality drone image products. There are several commercial (e.g., Pix4D, www.pix4d.com) and open-source (e.g., OpenDroneMap, www.opendronemap.org) software available for this purpose. As described above, different correction methods can be applied as needed depending on the environmental conditions during image collection and the sensors used, but the standard steps towards the final product are given below. For a more detailed description, see the different software manuals.

1. Orthorectification, i.e., a process that corrects the images by removing optical distortions related to the sensor, view angle, orientation, terrain, etc., so that it corresponds accurately to its geographic location.
2. Stitching the series of overlapping images into one composite orthomosaic.
3. Georeferencing of the orthomosaic, i.e., assigning real-world coordinates (latitude and longitude) to each pixel in the mosaic. This ensures that the image can be accurately positioned within common geographic information systems (GIS). Ground control points (GCPs) can be used in this process (see Georeferencing).

### Collection of ground-truth data

Collection of high-quality ground-truth data is essential for image annotation and subsequent training of machine learning (ML) algorithms, and to validate, i.e., determine the accuracy, of final habitat mapping products. Typically, ground-truth data are acquired *in situ* using traditional marine habitat mapping methods, but somewhat modified for drone image validation, by experienced field personnel familiar with the various species and habitat types in the area.

#### Planning phase

When planning the fieldwork, the area of interest should be thoroughly inspected to get an overview of the bathymetry and other important environmental gradients, such as waves and currents, which influence the natural distribution of the species in the area. To ensure all dominant species and habitats are covered, transect lines could follow these gradients, from shallow to deep, sheltered to exposed, or similar. Consulting existing aerial photos, e.g. satellite images, air photos or drone photos, can be useful for this task. If available, existing data on species and habitats can be integrated, e.g., from GBIF (www.gbif.org) or other global, national, or local data sources.

Prepared field maps (digital or paper), with transect lines assigned, should be brought to the field. Areas where the water is too deep to walk and where a boat is needed for ground truth data collection, can be indicated in the maps. As for the drone image collection, environmental and weather conditions should be considered. If there are great tidal differences in the survey area, ground-truthing during low tide is beneficial to have easier access to deeper areas. There is no need to collect ground truth data in areas that are too deep for the drones to “see”, however, this is not always possible to determine beforehand as the drone images may contain information at greater depths than what is visible with the naked eye in the field, depending on the optical sensor and water column properties.

To achieve the best possible match between drone images and ground-truth data, it is preferable to collect ground-truth data either simultaneously or in close temporal proximity to the drone flights. Coastal habitats are dynamic and are continuously transformed by the influence of currents, waves, and other factors (see Fig. 4 and the section Environmental conditions). As time passes, the potential discrepancy between the drone imagery and ground truth increases. In some cases, changes in tidal water, as well as solar elevation, could make it more relevant to ground truth the next day at the same time rather than hours after the drone flight.

#### What should be mapped?

Coastal habitats consist of biotic features such as different types of marine flora (e.g., tidal marshes, seagrass beds, rockweeds, and kelp forests) and fauna (e.g., mussel beds and barnacle encrustations), and abiotic features such as sand, and rocks of different sizes (pebble, cobble, boulder, and bedrock). Also, anthropogenic constructions and traces can often be identified in coastal images (e.g., boats, piers, houses, and marine litter). Identifying all these elements is important to get a high-quality classification map.

The level of detail at which ground-truth data should be recorded depends on the aim of the study and the quality of the drone images (i.e., image resolution and sensor type). E.g., if the aim is to separate marine vegetation from bare rock or sandy sediment, ground-truthing can be done by lumping all types of vegetation into one or a few classes, and similarly for the abiotic features. However, if one aims to achieve the most detailed habitat map possible or describe the biodiversity and its spatial cover at the species level, ground-truthing should be done on a systematically more detailed level. Still, there is no use in registering objects that are too small to be visible in the images. As a rule of thumb, objects in drone images can be recognized and classified down to a size of approximately four times the pixel width [15].

Using drone-based imaging and subsequent ML models, seagrass, which is a marine vascular plant with a strong green signal, can be separated from brown macroalgae, which has different pigmentation and textual structure [13,45]. The spectral variation between species can furthermore allow ML algorithms to distinguish between habitat groups and species from MSI and HSI data [17,46,47]. Thus, ground-truth data should preferably be collected at species level. Observations can later be merged to a coarser systematic level, if the drone data do not support differentiation on species level or if this is not the objective of the study (see Method validation and case study for an example of a hierarchical habitat class structure).

#### Sampling resolution

As the main aim of collecting ground-truth data is to guide the image annotation, and the accuracy of the data determines how directly they can be used in the annotation procedure, ground-truth data should be collected with a known geospatial accuracy, ideally matching the accuracy of the drone data. For instance, a regular “off the shelf” handheld GPS has a coarser spatial accuracy than drone images collected with RTK technology (spatial resolution of ∼2 m vs. a few cm, Fig. 3, Table 1). In these cases, ground-truth data can only guide the annotation. On the contrary, using high-precision GNSS location technology, with centimeter-scale accuracy (Table 1), will aid the annotation procedure significantly, as ground-truth observations can be overlayed accurately on top of the drone image and annotation can be done by drawing a polygon around the point (Fig. 3).

It is worth remembering that small patches, and the edges of larger patches, might move with winds, waves, and currents (Fig. 4). Thus, patches should be of a certain size and be positioned with a buffer around a point observation, and the accuracy of the georeferencing should be known. We suggest a minimum patch size of five times the pixel resolution of the image in the intertidal, and larger when mapping under water. For instance, if the pixel resolution in the drone image is 2 cm, then the patch should be >10 cm in diameter.

#### In situ data sampling

Ground-truth observations can be made by walking in the intertidal and shallow water, or from a boat in deeper waters (Table 2). If available, a high-resolution GNSS antenna should be used as far as possible without exposing the equipment to water (typically to around 1 m depth, or as far as you can walk steadily into increasingly deeper water).

**Table 2:** Summary of recommended methods, equipment, and the spatial accuracy of the resulting point observations, when sampling ground-truth data at different water depths.

Water depth	Method	Positioning system	Precision
< 1 m (or as far as you can walk)	Direct observations by walking with wader pants or wet/dry suit	GNSS antenna	< 3 cm, often better
1 – 5 m (or as far as you can see the seafloor)	Direct observations by naked eye or aquascope from a boat	Handheld GPS	2-5 m
5 – 10 m (or as far as the drone can “see”)	Observations using underwater camera or ROV from a boat	Handheld GPS	∼10 m

When it gets too deep for walking, ground-truthing can be done from a boat using an aquascope, or an underwater camera or small remotely operated vehicle (ROV). The boat's movement will likely decrease the accuracy of the collected data's georeferencing, and when using a cabled underwater camera or ROV, the positions’ accuracy decreases with the length of the cable and the cable's radius around the boat [48]. Due to this decrease in accuracy, it is often sufficient to use regular GPS instruments rather than high-precision GNSS systems when collecting ground truth data from a boat.

Observations of all habitat classes present in the study site (and captured in the drone images) should be recorded in a field form with associated waypoints (GPS coordinates). This can be done on pre-printed waterproof paper sheets with columns for location (waypoint), date and time, depth, observed habitat class, and other useful information (see Table S1 in the Supplementary material for an example). Alternatively, one can use a tablet or similar device. Some GNSS equipment even includes pre-programmable drop-down list functions for directly entering the habitat class when recording a waypoint. This feature minimizes the time required for punching data after the field work and reduces the risk of human error when noting waypoints or habitat classes in the field. When using field forms separate from waypoints, it can be useful to regularly photograph some of the waypoints to ensure correct linkage to the corresponding habitat class.

Unlike when collecting data for statistical distribution models, there is no need for either a strictly randomized or balanced dataset, if (1) the variation in the image dataset is covered, (2) the annotation dataset ends up relatively balanced, and (3) with all relevant classes represented with enough data points. In general, the performance of ML algorithms like CNNs will improve when adding more annotation data, but the number of ground-truth observations needed to perform the annotation will be case-dependent. In general, more ground truth observations may be needed for classes that are difficult to identify in the drone images, for example intertidal species that may change color, shape, and texture, depending on the tidal phase (Fig. 4), compared to classes that are easily detected from the drone images such as patches of sand.

### Image annotation

Annotation is the process of labeling drone images to identify and categorize different elements of interest (i.e., objects or segments). Accurate annotation provides the basis for subsequent ML analyses that translate the pixelwise drone images into habitat classes (see Image classification). During annotation, habitat classes found in the images are identified and marked as polygons, using ground-truth data for guidance. The identified polygons of habitat classes are stored in georeferenced vector datasets that are used as training samples for the ML algorithm.

#### Establish a GIS project

The georeferenced orthomosaics (raster files) and ground-truth dataset (point vector layers) are loaded into the annotation software (e.g. ArcGIS, QGIS). All available georeferenced images from different sensors (RGB, MSI and HSI) can be overlaid and used in the annotation process, but since RGB images show the natural color of the vegetation and usually have the highest resolution, they often provide the best starting point for annotation. Depending on the features of interest, it may also be helpful to calculate additional bands from the raw sensor data. For example, the Normalized Difference Vegetation Index (NDVI) or Normalized Difference Water Index (NDWI) may be helpful in some circumstances (e.g. above water and in the intertidal zone). False colors can be applied to MSI bands to exaggerate features of interest.

#### Define classes of interest

For consistency, it is helpful to pre-define the target classes of interest and, optionally, agree on a standardized color scheme for annotated classes. This information can be stored as either JSON or XML and loaded into the annotation software, which saves time compared to defining classes manually and ensures that everyone involved in the annotation process uses the same class definitions.

Some software (e.g. ArcGIS Pro's Image Analyst) supports hierarchical class definitions, which are useful in an ecological context. Embedding elements based on, e.g., the taxonomic hierarchy (such as family > genus > species) into the class definitions allows for more flexible annotation and ML. Within this framework, ecologists annotating images should attempt to label each polygon they draw with the most detailed class label they can confidently assign, falling back to less detailed levels in the hierarchy where necessary. This approach generally leads to better quality training data, because users have the flexibility to choose an appropriate label with confidence, rather than feeling pressured to assign specific labels despite high uncertainty.

Hierarchical annotation also provides more options for ML, because the labels can be used to generate multiple training datasets with different levels of detail. E.g., it is possible to begin by training a species-level classifier and then, if performance is not adequate, switch to coarser-level classifiers, without requiring any additional annotation work.

#### Create regions of interest (ROIs) and subareas

Usually, the orthomosaic covers areas that are not of interest, for example large areas of terrestrial habitat, human infrastructure, or deep water. In these cases, one should create a vector polygon defining the region of interest (ROI), excluding irrelevant areas and classes that are missing from the training samples.

In some cases, it may be worthwhile to further subdivide the ROI into sub-areas, which are annotated separately. This makes it easier to collaboratively annotate large images (e.g. each person agrees to annotate one sub-area), and it can provide a simple cross-validation scheme for ML. A disadvantage of this approach is that differences between annotated sub-areas may be due to “observer bias” (i.e. where different people have interpreted the image differently), rather than genuine ecological variation. This is not usually a problem for image classification, if the algorithm is trained on data generated by multiple annotators. Nevertheless, annotation bias resulting from observer bias poses a challenge for machine learning just as it does for classical statistics. For some datasets – especially those with obscure or hard-to-identify classes of interest – it may be worth having several people annotate the same (sub-)area. This gives an indication of the uncertainty in the annotation data used for model training, which in turn sets an upper limit on the performance that can be achieved by the ML model.

#### Identify habitat classes

Within the ROI, one starts the annotation process by drawing polygons around clearly distinguishable and identifiable habitat patches or objects. Here, the ground-truth data come into use. Keep in mind that high-precision observations can be used “as is” by drawing polygons around the point, while low-precision observations only can be used as guidance, accounting for the uncertainty in the accuracy of the location (see Sampling resolution). It may be useful to create an “uncertainty buffer” as a polygon layer around each low-precision point for this purpose. If using a hierarchical classification scheme, annotations should be assigned at the lowest (i.e. most detailed) level possible (see the case study below and github.com/SeaBee-no/annotation for examples of hierarchical classifications for coastal habitats).

While annotating, keep in mind that ML algorithms tend to work better with “balanced” training datasets i.e., where there are approximately equal numbers of samples in each class. This will often not be feasible when annotating drone images, but the dataset should nevertheless be as balanced as possible. This typically means spending more effort identifying and annotating uncommon classes, compared to more obvious ones. Annotated training samples can be saved as vector polygons, together with the ROI and sub-areas (if relevant). All vector layers should use the same coordinate reference system as the original image mosaic. In preparation for the image classification, it is convenient to gather all relevant layers, for example in a geopackage or geodatabase.

### Image classification

Object-based and pixel-based image analysis are two approaches for thematic mapping in images. For habitat classification, pixel-based image analysis is typically used, as one aims to classify all parts of the image into different habitat classes, in contrast to identifying specific objects.

#### Machine learning analyses

Deep learning (DL) has revolutionized the field of image analysis and is currently state-of-the art for solving a wide range of remote sensing image analysis tasks [20]. Specifically, deep CNNs consist of layers of convolutions that are applied directly to the input images and tailored to perform end-to-end analysis of the image data, e.g., pixelwise classification and parameter retrieval (see e.g.,[49]). Contrary to most conventional methods, CNNs do not only use single-pixel information to perform the analysis, but also the pixel values around the pixel of interest [50]. The CNN models can therefore learn to recognize textures, complex shapes, and spatial arrangement of colors to solve the task it is being trained to. One of the most popular CNNs for pixelwise classification is the U-Net architecture, which was originally developed for medical images [19], but has later been applied in a range of settings (see [51] for a detailed description and application to marine echosounder data).

In brief, the U-Net is a pixel-wise image segmentation network (i.e., a classification method) with a convolutional encoder–decoder architecture that can represent pixel-wise and abstract features simultaneously. The decoder takes these features and generates (decodes) an output for the different classes. The architecture also copies the lower-level features at each step when decoding, so that the decoder has access to both low-level features (e.g., the input bands in a small region) and more abstract features (e.g., the overall shape). Finally, the output is passed through a “softmax” function where each class is mapped to the interval [0, 1] and summing to 1. For pixelwise classification, the parameters in each convolutional layer must be estimated, which is done by defining a loss function measuring the loss between the predicted output and the annotated data. For classification, the cross-entropy loss function is the most common. We aim to minimize the loss function using “backpropagation”, i.e., the derivative chain rule from calculus solved iteratively using stochastic gradient decent [52].

Neural networks, like U-Net, often consist of millions of parameters, resulting in computationally intensive training. Consequently, deploying specialized computational resources, such as graphical processing units (GPUs), is often necessary to effectively handle this complexity. Moreover, the data-driven nature of neural networks, which aligns with the archetype of an ML approach [53], poses certain challenges. First, the network is sensitive to the balance in the training data: If the annotated data is dominated by one or a subset of the classes, the loss function may be minimized by just being able to predict the dominant classes, while the network may perform poorly on the less common classes. Second, the network learns the appearance of the data strictly from the training data, and may therefore perform poorly on new data from different areas, different light conditions, different times of the year, etc. Thus, to teach a model to work with sufficiently high performance on new image data, the annotated data should cover the expected variation in nature (species and habitats), physical conditions (water depth and optical properties), light conditions (solar elevation and latitude), image quality (sensors settings and specs), etc. Hence, as new ground truth data are collected and images annotated at new sites, they could with large benefits be added to an accumulating database of training data used to continuously improve the specific model. Ultimately, this would reduce the need to collect ground-truth data and perform image annotation in regions and under conditions that are similar to what has already been mapped.

#### Model validation and uncertainty characterization

When training an ML model, we typically divide the dataset into three parts: training, validation and test data, which must be clearly spatially separated [51]. The training dataset is used to compute the gradients in the backpropagation algorithm and learn the weights in the network, the validation dataset is used to assess the performance during training and select parameters like learning rate and batch size, and the test dataset is used to assess the performance of the final model. Typically, the training dataset constitutes a large part of the dataset (e.g., 60-80 %), whereas the validation and test datasets each constitute 10-20 %. All classes must be present in the training dataset, and for a fair performance evaluation they should also be represented in the validation and test datasets.

In addition to performing habitat classification, the model should preferably detect samples that are out-of-distribution (samples that do not belong to the distribution the model has been trained for, ensuring robustness and reliability of the model) and assess the uncertainty. Uncertainties in the predictions are the accumulated effects from errors inherent to the measurements (sensor noise, motion noise, label noise), variability in nature, errors in the model architecture specification, errors in the training procedure, and errors caused by unknown data. We typically categorize prediction uncertainty into data uncertainty and model uncertainty. The data uncertainty cannot be reduced, whereas the model uncertainty disappears given enough data [54].

## Method validation and case study

Below, we illustrate a field campaign that employed the methods described in this protocol, from drone image acquisition and ground-truth data collection to image annotation and habitat classification.

### The SeaBee field campaign at Remøy, autumn 2022

**Fig. 5:**
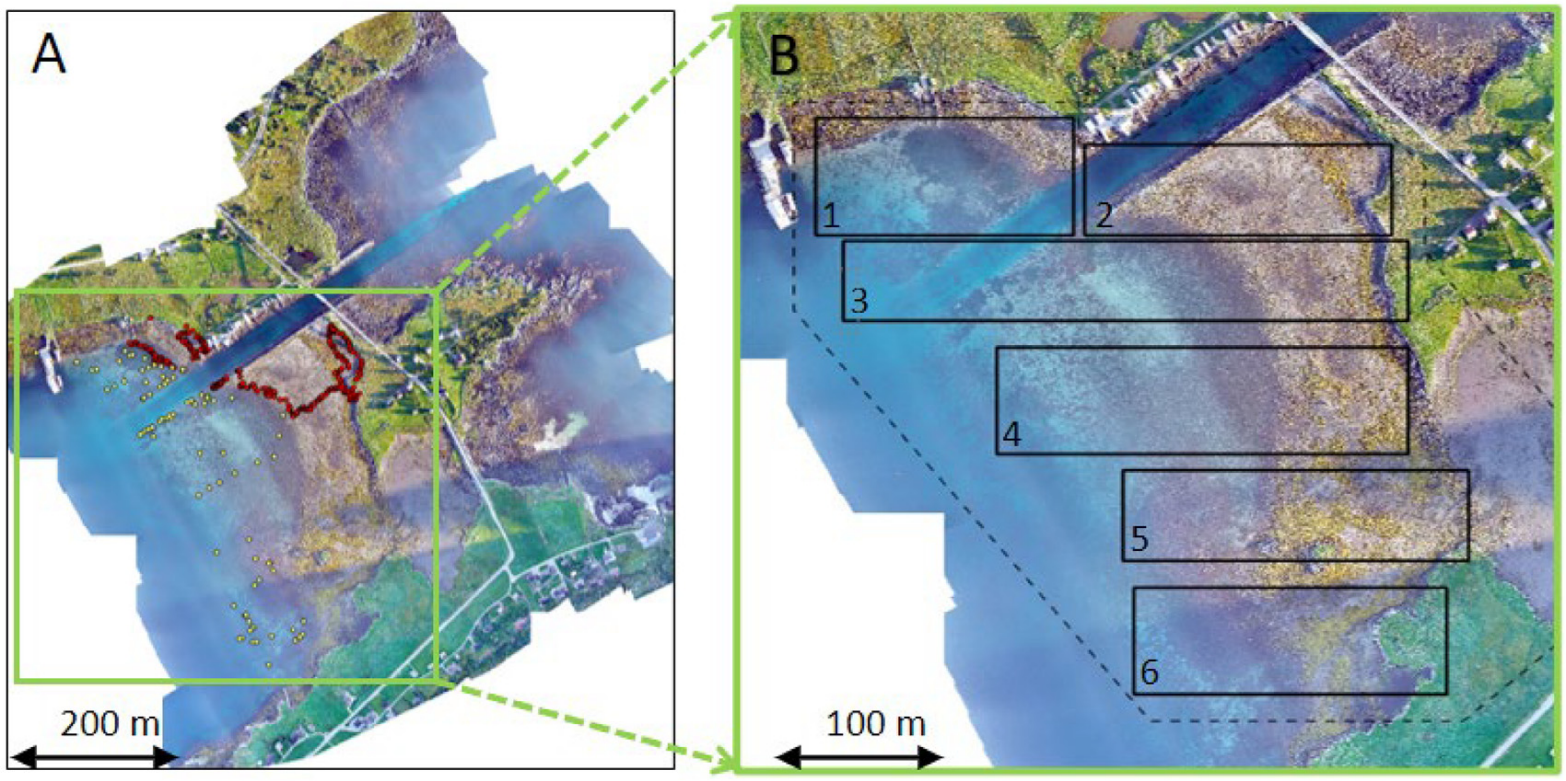
Overview of the Remøy study site. A) The complete orthomosaic composed from RGB imagery captured by drones, showing the locations of ground-truth data (red points: high-precision observations collected by walking in the intertidal and shallow areas; yellow points: less precise observations collected using boat in the subtidal). B) A zoomed in view of the region of interest (ROI) for image analyses (dashed box), and six distinct subareas used for image annotation and classification (1-6, solid boxes).

On September 1^st^ 2022, we conducted a field campaign in the coastal region between the Remøy and Leinøy islands in Herøy municipality, Møre and Romsdal County, Norway (approx. 62.36ºN/5.66ºE, Fig. 5). The field site encompassed intertidal and shallow subtidal zones dominated by marine vegetation (seagrass, seaweed/rockweed, and kelp), in addition to sandy sediments, gravel, and boulders. During the campaign, both drone imagery and ground-truth data were collected.

### Drone image collection

A total of five drone missions were conducted using a fixed-wing drone (eBee-X, AgEagle) with RTK module (Table 3). The timing of the flights followed the tide levels: Two missions were flown using an RGB camera (Aeria X, AgEagle), the first at low-tide and the second at high-tide. Two missions using an MSI camera (RedEdge-MX, MicaSense) were flown immediately following the RGB-flights, with one extra flight during mid-tide. All drone missions were flown under a clear sky (0 % cloud cover) with wind speed between 1 and 4 m/s (NE direction). Two of the flights had to undergo PPK workflow, using commercial HxGN SmartNet correction services, due to incomplete RTK fix during the flight. Individual images underwent orthorectification in Pix4D Mapper photogrammetry software and were mosaiced into high-resolution orthomosaics and reflectance maps providing final datasets with 2.9 cm and 9.4 cm resolution for RGB and MSI data, respectively.

**Table 3:** Overview of the drone missions completed September 1^st^ 2022, with time, sensor type, altitude, tidal level, flight time, area covered, number of acquired images, accuracy of the georeferencing of the orthomosaics (based on the RTK data), and Ground Sampling Distance (GSD, i.e., the processed image resolution).

Start time (GMT+01)	Sensor	Flight altitude	Tide	Flight time (min)	Area (ha)^1^	# acquired images	Accuracy (cm)	GSD (cm)
07:30	RGB	118 m	Low-tide	46	121.1	694	4.6	2.9
08:22	MSI	117 m	Low-tide	64	104.8	1502	4.7	9.3
10:43	MSI	117 m	Mid-tide	68	100.6	1513	4.9	9.4
13:09	RGB	118 m	High-tide	45	117.6	692	4.7	2.9
14:04	MSI	117 m	High-tide	67	101.7	1520	5.2	9.4

1 Mapped area following data processing in Pix4D Mapper.

### Collection of ground-truth data

**Fig. 6:**
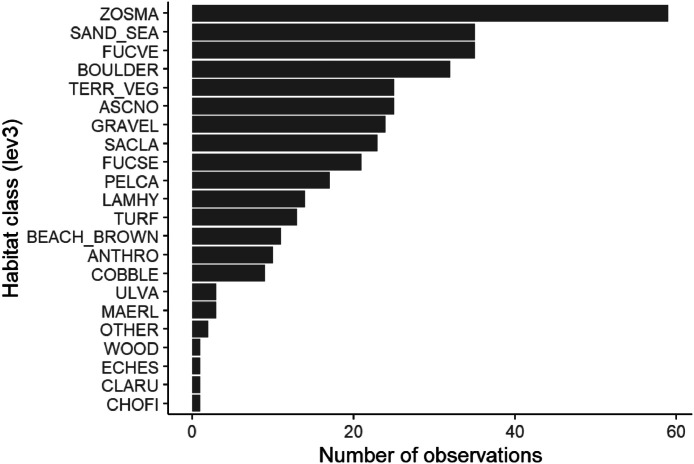
Number of ground truth data collected per habitat class (at the most detailed level). Note that the figure only shows the distribution of classes recorded at the exact locations of ground truth data (Fig. 5) and do not necessarily represent the classes’ relative abundance in the field.

At approximately the same time as the first drone flight (Table 3), we initiated the ground-truth data collection. We traversed transects that extended from land, across the intertidal zone, and towards deeper waters (Fig. 5). Equipped with waders and during a period of approximately 4 h, we recorded representative habitat classes and their associated waypoints using a high precision GNSS (Leica GS18T GNSS). At mid-tide, starting at approximately 17:30, we continued collection of ground-truth data in the subtidal zone from a boat, using aquascope, underwater camera and a handheld GPS (Garmin GPSMAP 66S). In total, we collected 407 ground-truth observations: 279 with high geographic precision in the intertidal and 128 with lower precision in the subtidal (Fig. 5). We aimed to record habitat classes in as much detail as possible, focusing on species of marine vegetation and different substrate types. The most frequently recorded species were the seagrass *Zostera marina*, the seaweeds *Fucus vesiculosus, Ascophyllum nodosum*, and *Fucus serratus*, and the kelp *Saccharina latissima* (Fig. 6). The most frequently recorded substrate types were sand, gravel, and boulders.

While we intended to cover the main environmental gradients, time constraints and limited familiarity with the survey area led to a ground-truth dataset skewed towards the northern subareas (Fig. 5) and dominant habitat classes (Fig. 6). Although imbalanced ground-truth data can be partially mitigated by emphasizing rare classes during annotation, the final training dataset for the ML algorithms still reflected this skewness, since some classes were underrepresented, and distinguishing algae species during annotation proved challenging (see below). Given that natural environments typically exhibits a few dominant species and numerous rare ones [55], achieving balanced training data in such studies is inherently difficult. For example, a recent systematic ground-truth sampling campaign in Northern Norway similarly yielded annotation data biased towards dominant classes due to the limited size and distribution of rarer species [39]. Still, we recommend careful planning of ground-truth sampling to maximize data coverage and achieve better balance.

### Image annotation

In ArcGIS Pro (v.3.0.3, www.esri.com), we defined a ROI that covered both the intertidal and subtidal habitats, as well as the full extent of the ground-truth data (Fig. 5). In doing so, we intentionally excluded most of the terrestrial areas and man-made infrastructure to focus on the marine environment. To facilitate a more structured annotation procedure, we further divided the ROI into six distinct sub-areas (Fig. 5).

When inspecting the orthomosaic, it became apparent that it was difficult to confidently distinguish between species of seaweed, such as *A. nodosum, F. vesiculosus,* and *F. serratus*, when relying solely on drone images. The high-precision ground truth observations proved to be very useful in these cases. However, for areas outside the exact locations of ground truth data, certain habitats could only be classified at a broader taxonomic level, rather than at species level. Consequently, we developed a hierarchical classification structure for habitat types: the first and most coarse level primarily distinguishes between vegetation and substrate; the second level differentiates the main types of macroalgae (brown, green, red), seagrass, and various substrate types such as boulder, cobble, and gravel; and the third and most detailed level differentiates between species (Table 4). The hierarchical classification structure builds largely on the established taxonomic hierarchy, can be re-used across field campaigns and locations, and provides a high degree of flexibility when training ML algorithms (see Define classes of interest). Note that, for some habitat classes, the coding remains the same across levels - for instance terrestrial grass, wood and anthropogenic materials. These classes were treated uniformly because they were not the focus of the field campaign.

**Table 4:** The hierarchical class structure used for habitat class annotation, based on data collected during the 2022 field campaign. The first three columns correspond to the codes used for annotation, from the most general (level 1) to the most specific (level 3). The final column provides the full species name for the third level or a description of the broader categories. See also https://github.com/SeaBee-no/annotation/.

1^st^ level	2^nd^ level	3^rd^ level	Species/habitat
ALGAE	BROWN	ALAES	*Alaria esculenta*
ALGAE	BROWN	CHOFI	*Chorda filum*
ALGAE	BROWN	LAMHY	*Laminaria hyperborea + L. digitata*
ALGAE	BROWN	PELCA	*Pelvetia canaliculata*
ALGAE	BROWN	SACLA	*Saccharina latissima*
ALGAE	BROWN	FUCSE	*Fucus serratus*
ALGAE	BROWN	ASCNO	*Ascophyllum nodosum*
ALGAE	BROWN	FUCSP	*Fucus spiralis*
ALGAE	BROWN	FUCVE	*Fucus vesiculosus*
ALGAE	BROWN	HALSI	*Halidrys siliquosa*
ALGAE	BROWN	DESAC	*Desmarestia aculeata*
ALGAE	GREEN	CLARU	*Cladophora rupestris*
ALGAE	GREEN	ULVA	*Ulva* spp, e.g., *U. intestinalis*
ALGAE	RED	PORPHYRA	*Porphyra* spp, e.g., *P. umbilicalis*
ALGAE	RED	CHOCR	*Chondrus crispus*
ALGAE	RED	FURLU	*Furcellaria lumbricalis*
ALGAE	RED	PALPA	*Palmaria palmata*
ALGAE	RED	VERLA	*Vertebrata lanosa*
ALGAE	TURF	TURF	Unspecified filamentous algae
ANGIO	ANGIO	ZOSMA	*Zostera marina*
MAERL	MAERL	MAERL	Maerl
URCHIN	URCHIN	ECHES	*Echinus esculentus*
URCHIN	URCHIN	STRDR	*Strongylocentrotus droebachiensis*
URCHIN	URCHIN	GRAAC	*Gracilechinus acutus*
STARFISH	STARFISH	OPHNI	*Ophiocomina nigra*
STARFISH	STARFISH	ASTRU	*Asterias rubens*
MUSSELS	MUSSELS	CRAGI	*Crassostrea gigas*
MUSSELS	MUSSELS	MYTED	*Mytilus edilus*
BEACHCAST	BEACHCAST_BROWN	BEACHCAST_BROWN	Beachcast, dried seaweed
BEACHCAST	BEACHCAST_ANGIO	BEACHCAST_ANGIO	Beachcast, dried seagrass
BEACHCAST	BEACHCAST_UNSPEC	BEACHCAST_UNSPEC	Beachcast, unspecified
TERR_VEG	TERR_VEG	TERR_VEG	Terrestrial vegetation
WOOD	WOOD	WOOD	Various wood incl. driftwood
ROCK	BEDROCK	BEDROCK	Bedrock
ROCK	BOULDER	BOULDER	Boulder
ROCK	COBBLE	COBBLE	Cobble
ROCK	GRAVEL	GRAVEL	Gravel
SEDIMENT	SAND	SAND_SEA	Sandy seafloor
SEDIMENT	SAND	SAND_LAND	Sandy beach
SEDIMENT	MUD	MUD	Muddy seafloor
DEEP	DEEP	DEEP	Deep sea (deeper than Secchi depth)
ANTHRO	ANTHRO	ANTHRO	Anthropogenic, e.g., house, litter, plastics

**Fig. 7:**
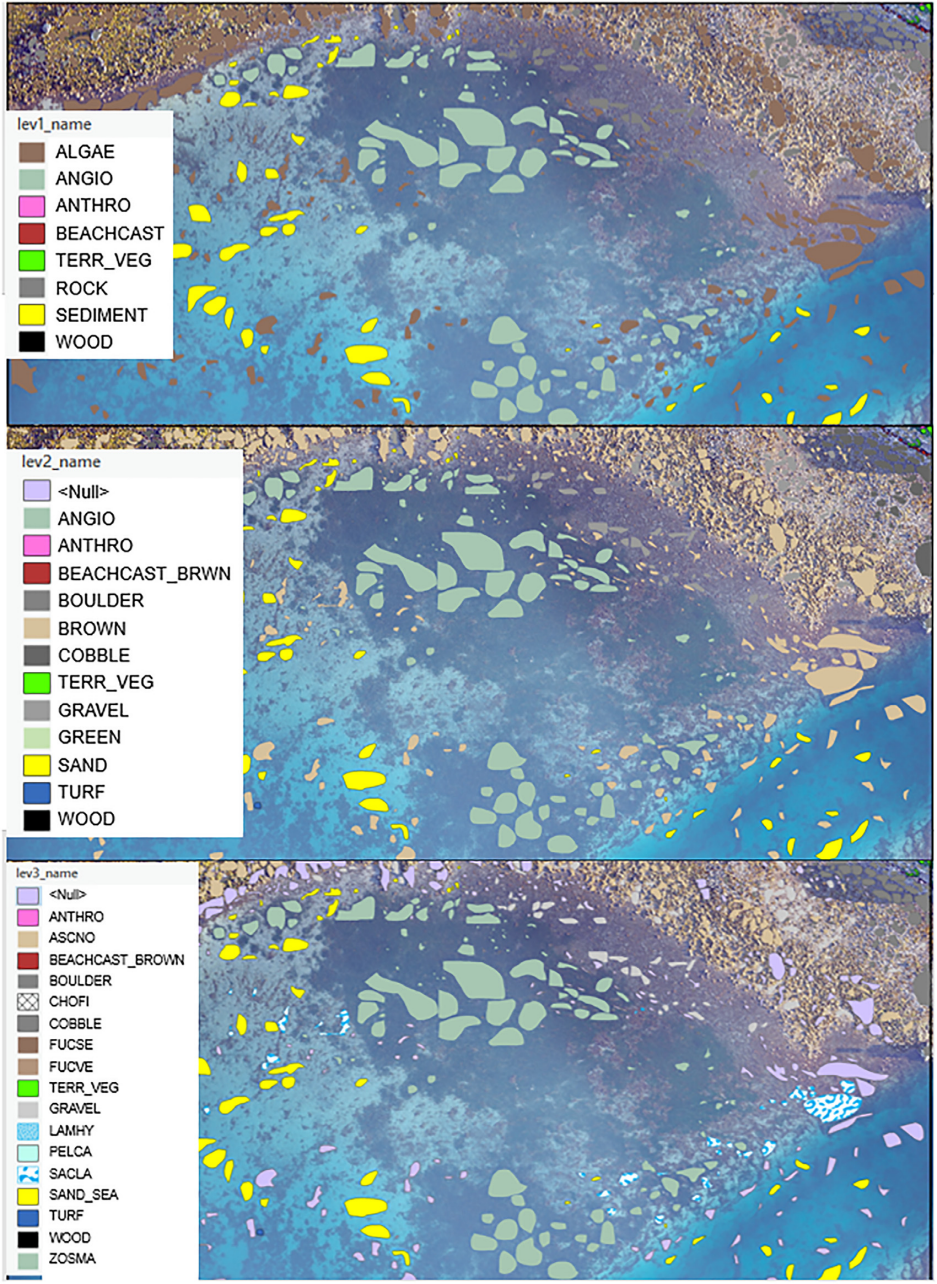
Example of annotations made for subarea 1 (Fig. 5), displayed at the three different levels in the habitat class hierarchy (Table 4). Polygons labeled as <Null> indicate that annotation was performed to a coarser level than the level displayed (typically brown algae). See Figure S1 in the Supplementary material for illustrations from the other subareas.

Annotations were digitized using ArcGIS Pro's Training Samples Manager, found within the Classification Tools of the Image Analyst extension. This software supports hierarchical class definitions, which can be defined manually via the Training Samples Manager or loaded as an ESRI Classification Schema (JSON) file. We performed annotation on the RGB image shown in Fig. 5, focusing on the six distinct subareas within the defined ROI and using the ground-truth data for guidance. Fig. 7 illustrates the annotations for subarea 1 (upper left in Fig. 5), which are divided into the three levels of detail. See the Supplementary material for illustrations from the other subareas. Note that the annotation for levels 2 and 3 includes polygons labelled as “Null”, which correspond to polygons that could only be assigned labels at less detailed levels of the hierarchy. This is typically the case for categories like brown algae, which were not distinguished at species level. See also https://github.com/SeaBee-no/annotation/ for a detailed workflow and downloadable files for habitat class definitions.

### Image classification

**Fig. 8:**
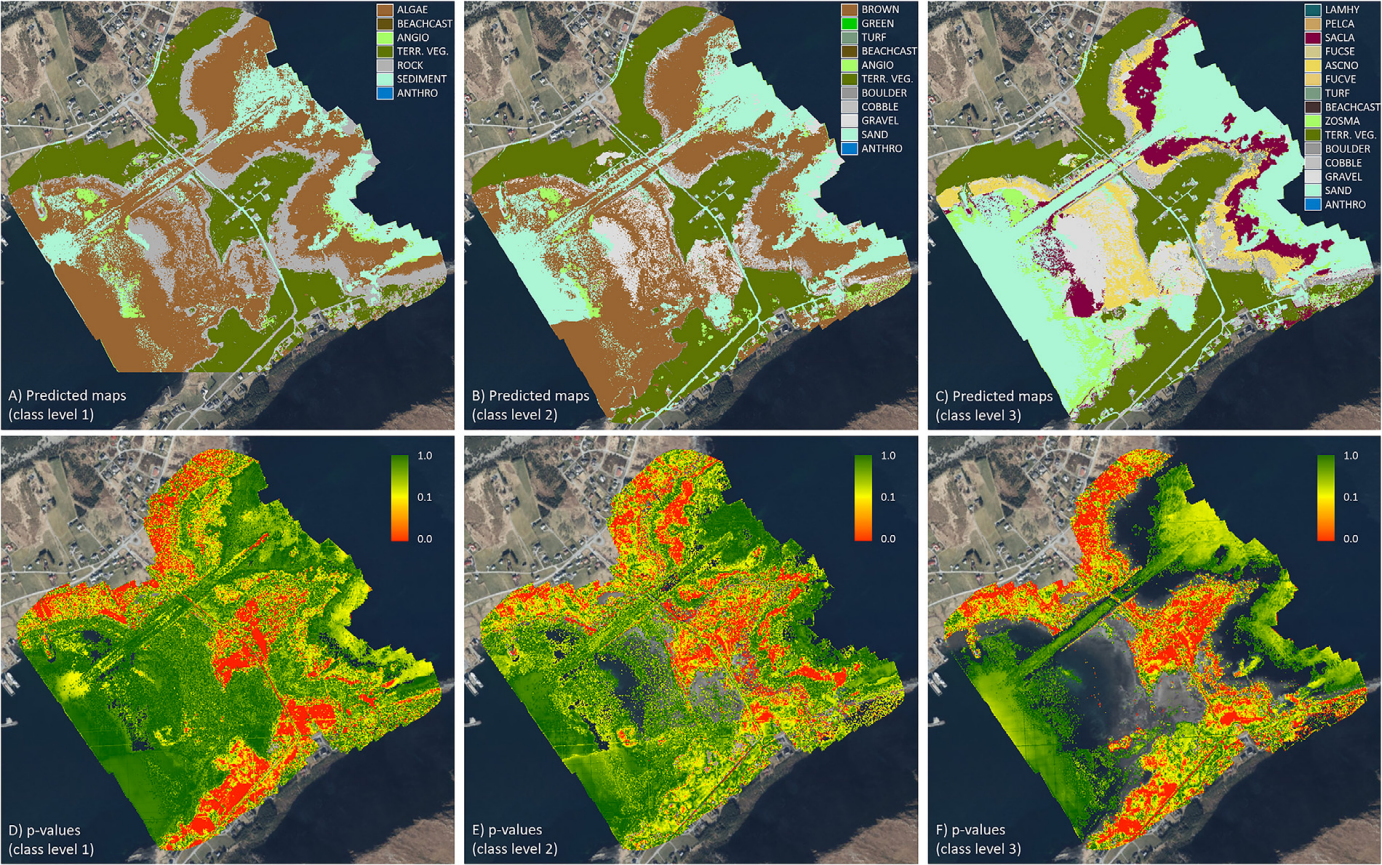
The final results of the coastal habitat classification at the case study field site, based on drone imagery and ground truth data collection, image annotation, and machine learning classification. A-C illustrates the habitat classification per pixel for separate model runs at the three different levels of detail in the habitat class hierarchy (Table 4). D-F: p-values associated with the classification per pixel. Higher p-values (green colors) indicate higher confidence in the predicted habitat class, while low p-values (red colors) indicate lower confidence (the null-hypothesis is to accept the predicted habitat class). See text for details.

Pixelwise habitat classification was performed using the U-Net model on MSI images collected at low tide (Table 3), following Liu et al. 2022 [17]. We ran the model on each level of the hierarchy separately, resulting in three maps where each pixel is classified to one of the classes in the respective hierarchy, and each pixel-based classification is associated with a level of confidence (i.e., p-value, Fig. 8). The model was trained on subareas 1-4 (Fig. 5), while subarea 5 was used for validation, i.e. to evaluate performance during training and estimate parameters to calculate the p-values. The procedure for calculating the p-values is based on the distribution of the values before the softmax layer in the U-Net [56].

The complete habitat map presented in Fig. 8 represents the final product of the drone mapping mission, which is a shallow water coastal habitat classification. The classification map integrates the information from the *in situ* collection of drone imagery and ground truth data, the annotation process, and the final steps of training, validation and testing of the machine learning classification.

At the coarsest level (level 1), the habitat map separates the field site into mostly macroalgae and seagrass (classes ALGAE and ANGIO), soft bottom (class SEDIMENT) and rock, as well as terrestrial vegetation (Fig. 8). Level 2, in turn, separates the different types of rock, gravel being the most dominant, while also suggesting a larger fraction of sandy bottom relative to macroalgae cover compared to level 1 (note that the three hierarchies are modeled independently). At the most detailed level (level 3), the habitat map shows the distribution of seaweed species in the shallowest areas (classes FUCSE, FUCVE and ASCNO) and kelp (SACLA) in areas with intermediate depth. Here, the deepest areas are predicted to be mostly sandy bottom, however, the predictions are associated with higher uncertainty.

The habitat classification performed well across the two first levels of detail in the habitat class hierarchy, with generally high p-values suggesting high confidence in the predicted habitat classification for a given pixel (note that the null-hypothesis is to accept the predicted habitat class). The p-values can potentially be used to mask out areas of the map where the classification is highly uncertain (masking pixels with p-values below a certain threshold, e.g. <0.05). In our case, this would mean that terrestrial areas, which were outside the scope of the campaign and for which the amount and variation of training data created during the annotation procedure was lower, would largely be masked out. The gaps in the p-value maps, especially apparent for level 3, are due to some of the habitat classes missing in the validation data set. In general, the performance of the classification for level 3 was reduced due to 1) all habitat classes not being represented across all sub-areas in the training data, 2) the training data being quite unbalanced, and 3) limited annotation data for many of the classes. The sensitivity of ML algorithms to these factors is important to consider when annotating drone images, as it may limit the level of detail at which an acceptable habitat classification can be achieved.

## Summary and conclusions

The primary objective of the method described here is to identify and delineate shallow water marine habitats, including seagrass, kelp, and various other types of macroalgae, and distinguishing these biotic features from abiotic ones, such as sand, boulders, gravel, as well as terrestrial vegetation. Detailed and comprehensive mapping and classification can serve as a vital tool to understand and preserve ecologically valuable marine ecosystems and is essential to secure sustainable management actions. As human development in the world's coastal zones increases and we face the increasing pressures of a triple planetary crisis (climate change, biodiversity loss and pollution [57]), we urgently need systematic and cost-effective methods for ecosystem monitoring.

Using drone-collected image data offers a consistent and reproducible way to map and classify coastal areas more efficiently than with traditional methods. At the same time, drone imagery and habitat classification provide more detailed information (cm scale) than what is available from satellite remote sensing data (meter to tens-of-meters scales). Moreover, in contrast to satellites, drones allow for on-demand data collection in specific areas of interest at specific times, facilitating more frequent sampling. This makes drones equipped with optical sensors ideal for assessing temporal variation in spatial distribution, coverage and patchiness of coastal habitats, and for mapping dynamic species and habitats like blue forests at larger scales. Considering the fast technological development, it is likely that the methodology in the future can be used for assessment of blue carbon repositories, ecosystem health and status, and biodiversity.

This protocol is based on several years of experience within SeaBee and earlier projects, with many rounds of trial and errors, and subsequent evaluations. Still, several challenges remain. For example, we aim to scale up the areal coverage of drone missions while maintaining high resolution of the image output. This is associated with more complicated missions for the pilots (who also face an increasing number of regulations and local requirements), and immense volumes of data which need to be handled down efficient data pipelines. Another key aim is to obtain ML models which are reproducible across areas without the need of new labor-intensive ground truth and annotation work. This is challenged by the large variability of coastal habitats due to ecological state and status, different light conditions, tidal levels, water properties, etc. Newer ML models like vision transformers [58] and self-supervised learning should be explored in future habitat classification efforts. Hopefully, by establishing a foundation model [59] for coastal vegetation in drone images we can obtain high performance on image data from new locations by just annotating a small amount of data. Furthermore, annotation software is continuously improved, and annotation classification lists likely need to be updated when mapping in new regions, which both dictates regular updates of methodology and protocols that are applied. Finally, in comparison to the use of underwater cameras or scuba diving, images from flying drones are limited to shallower areas of the coast, although the steady technological development of optical sensors paves the way for increased depth penetration in water (e.g. [40]). Thus, while drone imagery cannot replace traditional methods completely for subsurface marine mapping, it offers a cost-efficient supplement, and, in concert with other new technologies like USVs and ROVs [60], much needed opportunities for digitalized and reproducible ecosystem monitoring and research.

## Ethics statements

This study does not involve any human subjects, animal experiments or data collected from social media platforms.

## CRediT authorship contribution statement

**Kristina Øie Kvile:** Conceptualization, Formal analysis, Investigation, Writing – original draft, Writing – review & editing. **Hege Gundersen:** Conceptualization, Methodology, Investigation, Project administration, Visualization, Writing – review & editing. **Robert Nøddebo Poulsen:** Data curation, Methodology, Investigation, Writing – review & editing. **James Edward Sample:** Formal analysis, Software, Methodology, Data curation, Writing – review & editing. **Arnt-Børre Salberg:** Formal analysis, Software, Methodology, Visualization, Writing – review & editing. **Medyan Esam Ghareeb:** Data curation, Methodology, Investigation, Writing – review & editing. **Toms Buls:** Data curation, Methodology, Investigation, Writing – review & editing. **Trine Bekkby:** Conceptualization, Methodology, Writing – review & editing. **Kasper Hancke:** Conceptualization, Methodology, Investigation, Funding acquisition, Project administration, Writing – review & editing.

## Declaration of competing interest

The authors declare that they have no known competing financial interests or personal relationships that could have appeared to influence the work reported in this paper.

## Data Availability

See github.com/SeaBee-no/annotation for detailed workflow and files for habitat class definitions. All SeaBee data products are publicly available at geonode.seabee.sigma2.no.

## Appendix Supplementary materials

Image, application 1
