# Supplementary material for: Drone and ground-truth data collection, image annotation and machine learning: A protocol for coastal habitat mapping and classification
Source: MethodsX. 2024 Aug 30;13:102935. doi: 10.1016/j.mex.2024.102935 (PMC11409010; doi:10.1016/j.mex.2024.102935)
Supplement: Supplementary file 1 [file mmc1.docx]

**Drone and ground-truth data collection, image annotation and machine learning: A protocol for coastal habitat mapping and classification**

**Supplementary material**

*Table S1: Example of field form used for collection of ground-truth data. The blue text is example data from the Remøy field campaign in 2022.*

| **Area:** | **Remøy** | | | | *Survey area* |
| --- | --- | --- | --- | --- | --- |
| **Date:** | **31.08.2022** | | | | *Date of survey* |
| **Scheme #:** | **1** | | | | *Count of the current form for same area/date (1, 2 ...)* |
| **Name:** | **Kristina/Hege** | | | | *Person taking notes* |
| **Method:** | **Leica GNSS/direct observations** | | | | *Sampling method (e.g. GPS type, direct observation or camera)* |
| Waypoint | Time | Class code | Depth (m) | Patch ⌀ (cm) | Comment |
| **1** | **08:15** | **ASCNO** | **0** | **25** |  |
| **2** |  | **FUCSE** | **0** | **20** |  |
| **3** |  | **FUCVE** | **0** | **20** |  |
| **4** |  | **ZOSMA** | **0.3** | **50** |  |
| **5** |  | **ZOSMA** | **0.4** | **50** |  |
| **6** |  | **PELCA** | **0.4** | **25** |  |
| **7** |  | **PELCA** | **0.5** | **25** |  |
| **8** |  | **ZOSMA** | **0.5** | **50** |  |
| **9** |  | **GRAVEL** | **0.5** | **50** |  |
| **10** |  | **ZOSMA** | **0.5** | **50** |  |
| **11** | **08:30** | **SACLA** | **0.5** | **25** | **Young SACLA** |
| **12** |  | **TURF** | **0.5** | **20** |  |
| **13** |  | **SAND_SEA** | **0.6** | **20** |  |
| **14** |  | **SAND_SEA** | **0.6** | **50** |  |
| **15** |  | **SAND_SEA** | **0.6** | **50** |  |
| **16** |  | **SACLA** | **0.6** | **25** |  |
| **17** |  | **ANTHRO** | **0.5** | **25** | **Blue plastic litter** |
|  |  |  |  |  |  |
|  |  |  |  |  |  |
|  |  |  |  |  |  |


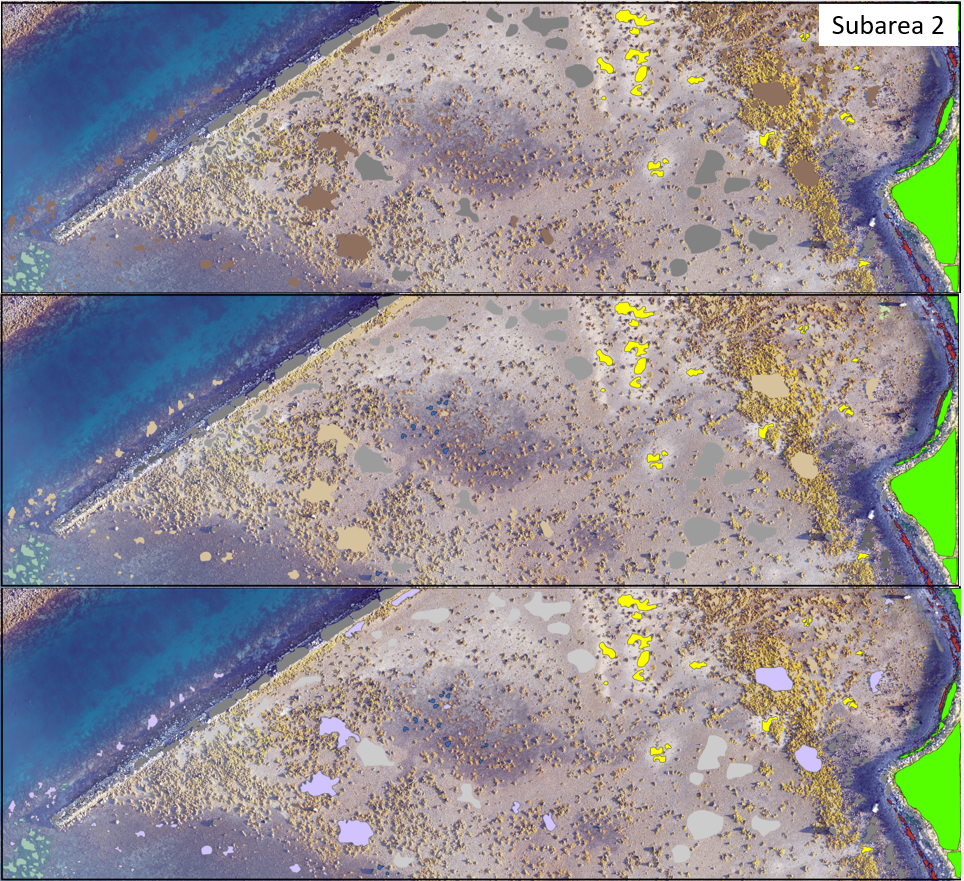

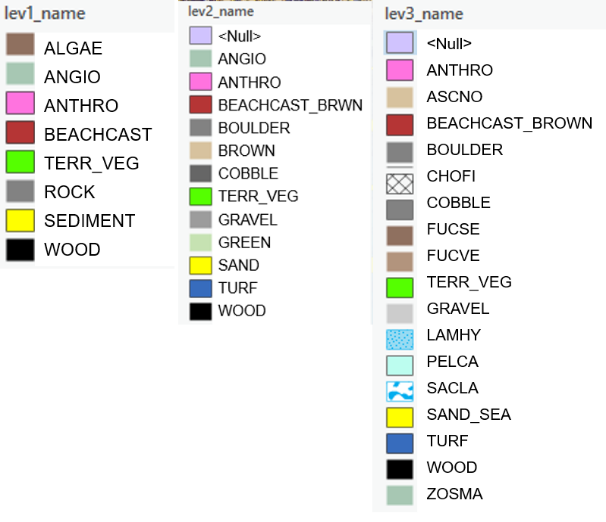


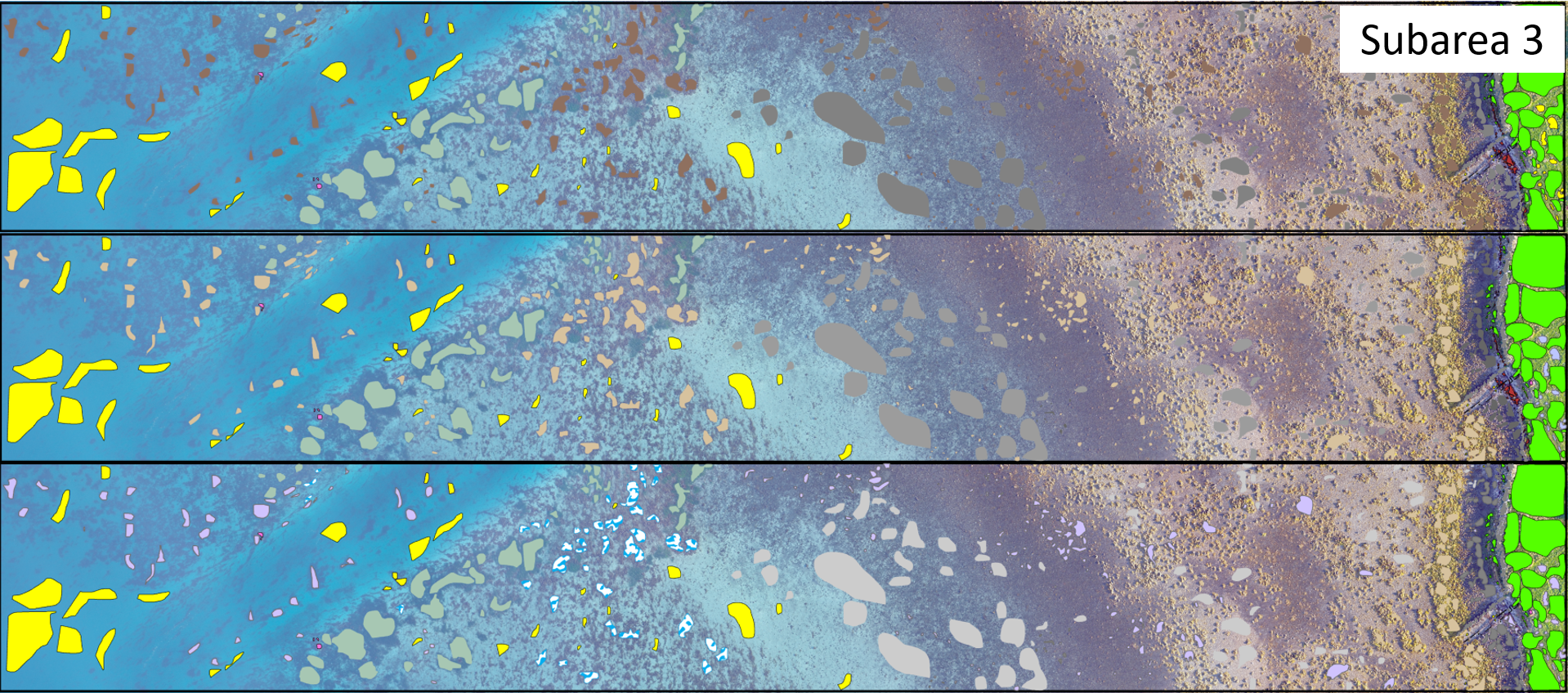

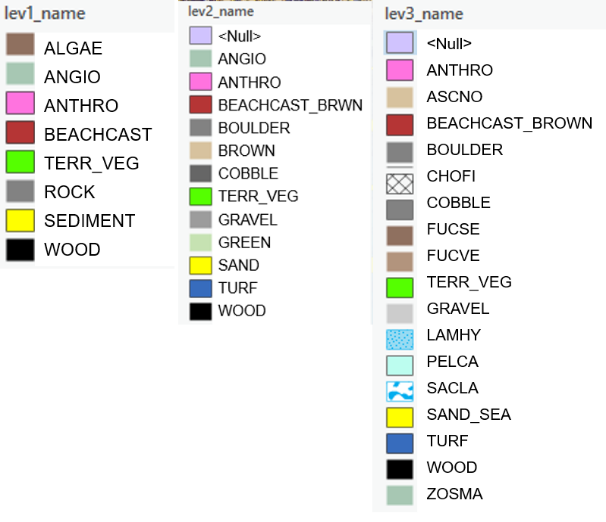


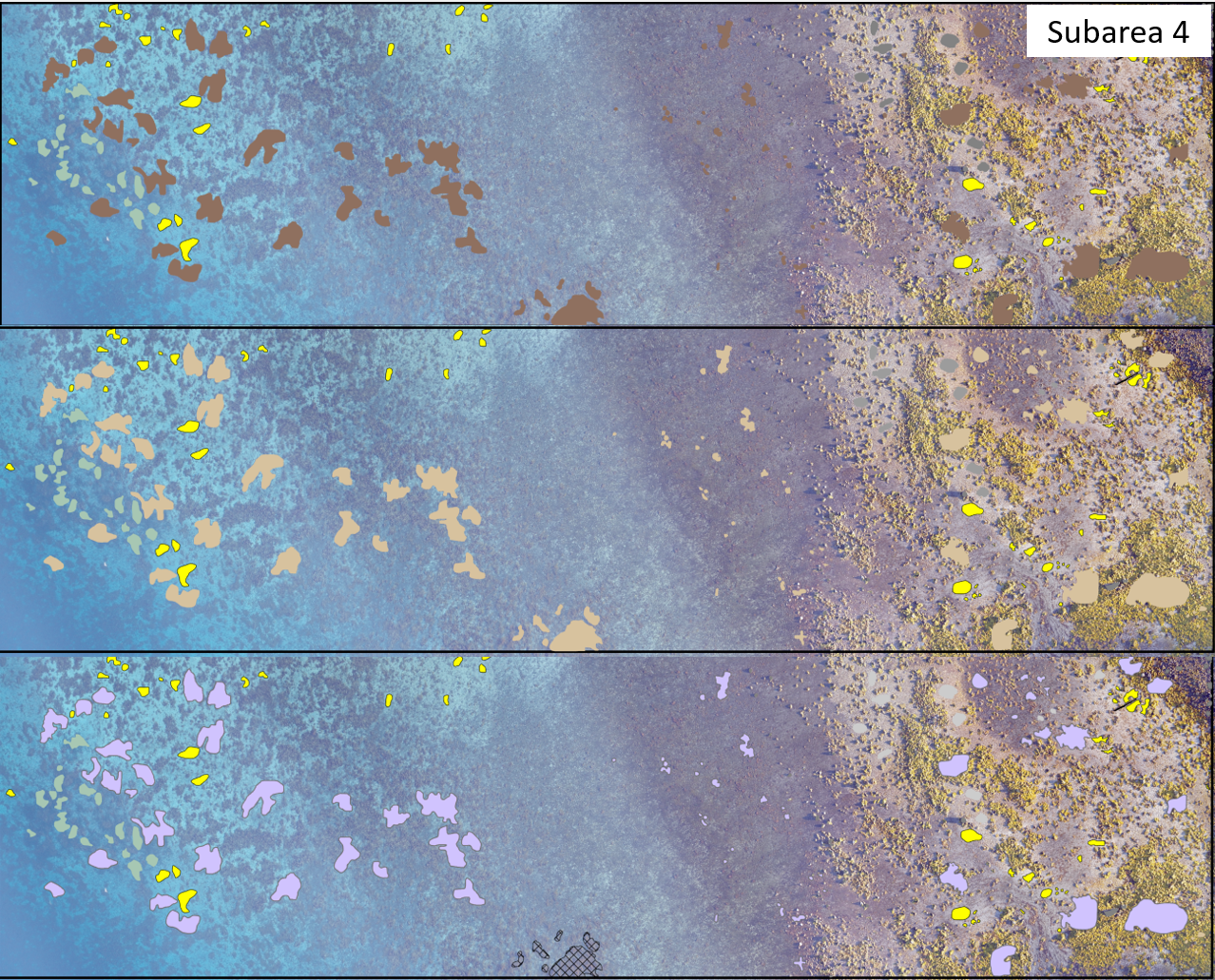

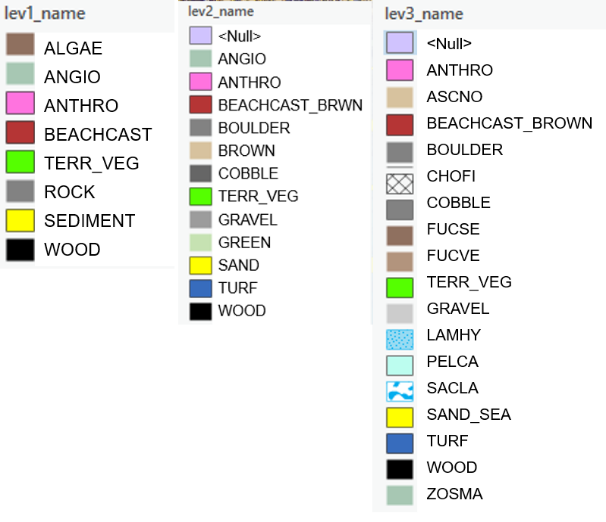


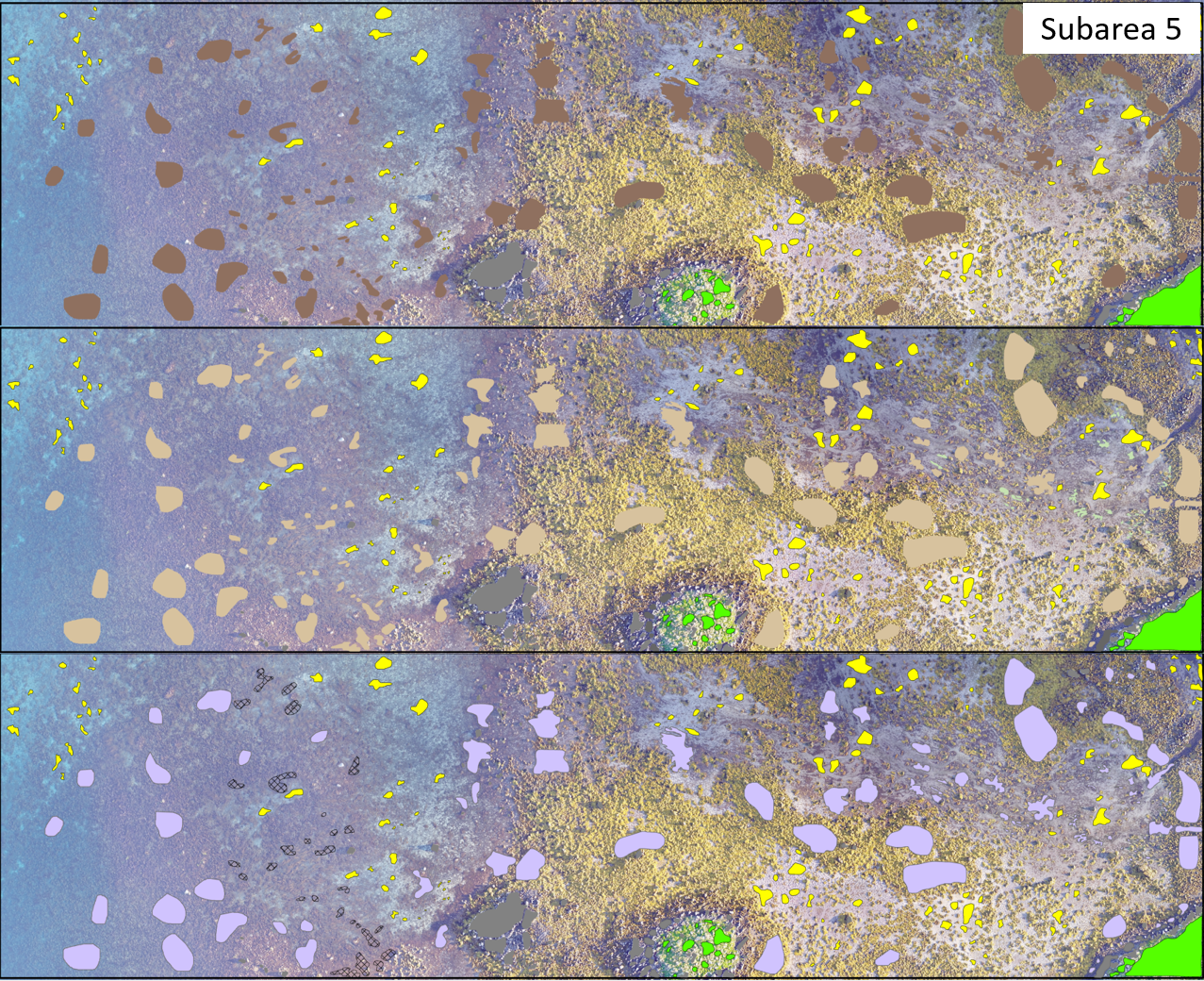

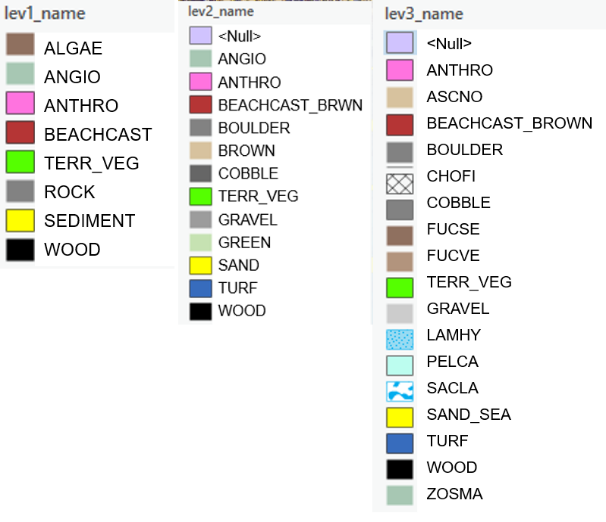


*Figure S1: Examples of annotations made for subareas 2-5, displayed at the three different levels in the habitat class hierarchy (from level 1 in the top row to level 3 in the bottom row). See Figure 7 in the main text for subarea 1 and Table 4 for a list of all the habitat classes. The U-Net model for pixelwise habitat classification was trained on subareas 1-4, while subarea 5 was used for validation. Polygons labeled as <Null> indicate that annotation was performed to a coarser level than the level displayed (typically brown algae).*
